# A Suicide Prevention Intervention for Emerging Adult Sexual and Gender Minority Groups: Protocol for a Pilot Hybrid Effectiveness Randomized Controlled Trial

**DOI:** 10.2196/48177

**Published:** 2023-09-29

**Authors:** Lily A Brown, Jessica L Webster, Jennifer T Tran, James R Wolfe, Jesse Golinkoff, Esha Patel, Amanda C Arcomano, Jennifer Ben Nathan, Alexander Azat O'Connor, Yiqin Zhu, Maria Oquendo, Gregory K Brown, David Mandell, Danielle Mowery, José A Bauermeister

**Affiliations:** 1 Department of Psychiatry Perelman School of Medicine University of Pennsylvania Philadelphia, PA United States; 2 Department of Family and Community Health School of Nursing University of Pennsylvania Philadelphia, PA United States; 3 Institute for Biomedical Informatics University of Pennsylvania Philadelphia, PA United States

**Keywords:** lesbian, gay, bisexual, transgender, queer, plus, LGBTQ+ health, suicide, peer navigator, emerging adults, life skills, mobile phone

## Abstract

**Background:**

Suicide attempts and suicide death disproportionately affect sexual and gender minority emerging adults (age 18-24 years). However, suicide prevention strategies tailored for emerging adult sexual and gender minority (EA-SGM) groups are not widely available. The Safety Planning Intervention (SPI) has strong evidence for reducing the risk for suicide in the general population, but it is unclear how best to support EA-SGM groups in their use of a safety plan. Our intervention (Supporting Transitions to Adulthood and Reducing Suicide [STARS]) builds on content from an existing life skills mobile app for adolescent men who have sex with men (iREACH) and seeks to target core risk factors for suicide among EA-SGM groups, namely, positive affect, discrimination, and social disconnection. The mobile app is delivered to participants randomized to STARS alongside 6 peer mentoring sessions to support the use of the safety plan and other life skills from the app to ultimately reduce suicide risk.

**Objective:**

We will pilot-test the combination of peer mentoring alongside an app-based intervention (STARS) designed to reduce suicidal ideation and behaviors. STARS will include suicide prevention content and will target positive affect, discrimination, and social support. After an in-person SPI with a clinician, STARS users can access content and activities to increase their intention to use SPI and overcome obstacles to its use. EA-SGM groups will be randomized to receive either SPI alone or STARS and will be assessed for 6 months.

**Methods:**

Guided by the RE-AIM (reach, efficacy, adoption, implementation, and maintenance) framework, we will recruit and enroll a racially and ethnically diverse sample of 60 EA-SGM individuals reporting past-month suicidal ideation. Using a type-1 effectiveness-implementation hybrid design, participants will be randomized to receive SPI (control arm) or to receive SPI alongside STARS (intervention arm). We will follow the participants for 6 months, with evaluations at 2, 4, and 6 months. Preliminary effectiveness outcomes (suicidal ideation and behavior) and hypothesized mechanisms of change (positive affect, coping with discrimination, and social support) will serve as our primary outcomes. Secondary outcomes include key implementation indicators, including participants’ willingness and adoption of SPI and STARS and staff’s experiences with delivering the program.

**Results:**

Study activities began in September 2021 and are ongoing. The study was approved by the institutional review board of the University of Pennsylvania (protocol number 849500). Study recruitment began on October 14, 2022.

**Conclusions:**

This project will be among the first tailored, mobile-based interventions for EA-SGM groups at risk for suicide. This project is responsive to the documented gaps for this population: approaches that address chosen family, focus on a life-course perspective, web approaches, and focus on health equity and provision of additional services relevant to sexual and gender minority youth.

**Trial Registration:**

ClinicalTrials.gov NCT05018143; https://classic.clinicaltrials.gov/ct2/show/NCT05018143

**International Registered Report Identifier (IRRID):**

DERR1-10.2196/48177

## Introduction

### Background

Suicidal behavior disproportionately affects sexual and gender minority youth [1] who are also at high risk for risk factors for suicide including harassment and trauma [2-4], yet few interventions address the specific needs of this population. In the United States, suicide rates increased for emerging adults (aged 18-24 years) in the past decade; suicide is now the second leading cause of death [5,6]. Youth who identify as sexual and gender minorities are >3 times more likely to have made a suicide attempt than those identifying as heterosexual [7]. Although sexual and gender minority individuals constitute only 4.5% of the general population (rates vary widely across studies), 10% of emerging adult (aged 18-24 years) suicide deaths occur among people who identify as sexual and gender minorities, representing a significant disparity [8]. Evidence-based suicide prevention programs tailored for emerging adult sexual and gender minority (EA-SGM) groups are not widely available.

Interventions to reduce suicidal ideation and behavior must address the unique risk factors that predict suicidal ideation and attempts in EA-SGM groups, including discrimination [9-11], family or friend rejection [9,12-16], and low positive affect [17]. Moreover, interventions promoting protective behaviors and social support may provide a complementary approach to traditional interventions to reduce risk in EA-SGM groups [18,19]. Developing youth’s assets (eg, coping strategies and activity scheduling to promote positive affect) and linkage to health-promoting resources (eg, social support and access to safe spaces) may reduce suicidal ideation. EA-SGM people who can envision a healthy future in which they achieve their long-term goals have, on average, great psychological well-being [20], few risk behaviors [21,22], and improved use of health services [23]. These findings are consistent with the suicide prevention literature, in which goal-action consistency is targeted to reduce suicide risk [24-26] and improve mood regulation [27,28], and social connection is viewed as a factor that reduces the likelihood of developing suicidal ideation [29,30] or the intensity of suicidal ideation [31]. Studies are yet to evaluate the extent to which improving coping with discrimination, promoting positive affect, and improving social support are associated with reduced risk for suicide among EA-SGM groups.

Individuals at risk for suicide and clinicians alike perceive Safety Planning Intervention (SPI) as acceptable and useful [32,33]. SPI is a brief intervention that combines evidence-based strategies to reduce suicidal behavior by providing prioritized coping strategies, identifying sources of social and professional support, and providing lethal means counseling to reduce access to potential suicide methods. Several studies have demonstrated that SPI can be successfully implemented in a variety of contexts, including university counseling centers [34] and crises call centers [35]. However, this intervention has not been explicitly evaluated among sexual and gender minority youth. This is important because there are many unique circumstances in some sexual and gender minority youths’ lives that may require adaptation of SPI, including reduced family support [36], experiences of discrimination [37], and low positive affect in general [38], each of which is a unique risk factor for suicide.

SPI and follow-up contact is associated with a significantly reduced risk of suicide attempts [39]; however, some studies indicate that individuals struggle to use the safety plan in suicidal crises. Patients reported barriers to using their safety plan after they generate it, which include the following: difficulty in finding the paper form; not thinking about or being motivated to use the plan during crises; and struggling with social isolation, which reduces the ability to enact safety plan steps to connect with one’s support network [40]. Recent studies have found that mobile apps, such as BeyondNow, Safety Net, and MYPLAN, show promise for implementing safety plans [41]. However, patients tend to discontinue using mobile apps in general, and among sexual and gender minority communities, a common sentiment is that apps are initially engaging but not necessarily effective [42]. To address this issue, we propose to bolster app engagement by providing users with weekly peer mentor sessions. The potential of this peer mentor care navigator approach in reducing suicide risk among lesbian, gay, bisexual, transgender, queer, plus (LGBTQ+) youth has not been evaluated.

EA-SGM people are early adopters of technology and rely heavily on e-delivered information [43,44], often ranking the web as their top resource to explore their sexuality and to access social support [45-49]. Our proposed intervention takes advantage of this phenomenon by examining whether a web intervention, Supporting Transitions to Adulthood and Reducing Suicide (STARS), reduces suicidal ideation among EA-SGM groups. We propose to examine a mobile app, STARS, which leverages peer mentoring and support to ensure that EA-SGM groups can implement their safety plan in times of suicidal crises. STARS will provide users with web opportunities to receive goal-related feedback and social support from clinically supervised peer mentors, who will function as care navigators. This peer-to-peer approach offers a confidential and user-controlled opportunity to support EA-SGM groups. We will assess whether the peer mentor model to provide social support reduces the impact of discrimination, promotes positive affect, overcomes barriers to SPI use, and decreases suicidal ideation.

### Objectives

STARS was developed based on the Integrated Behavior Model [50] and will include both the app and peer mentor access. Consistent with type-1 hybrid effectiveness randomized controlled trial design, our pilot study examines preliminary efficacy end points alongside implementation targets. We use the RE-AIM (reach, efficacy, adoption, implementation, and maintenance) framework [51] to guide the assessment of these concurrent end points. If successful, this pilot study will identify the potential clinical utility of STARS for suicide prevention in a vulnerable, often marginalized population based on key parameters needed to inform a future clinical trial.

Our study goals are (1) to develop STARS through a systematic adaptation of an existing web intervention (iREACH [52]) using the assessment, decision, administration, production, topical experts–integration, training, and testing (ADAPT-ITT) [53] framework; (2) to examine the preliminary efficacy (suicidal ideation and behaviors) and mechanisms of action of STARS, relative to our control condition (SPI only); and (3) to examine STARS’ implementation outcomes when compared with the control arm using RE-AIM [51,54] metrics. This exploratory project will identify the potential clinical utility of STARS for suicide prevention in a vulnerable, often marginalized population based on key parameters needed to inform a future efficacy trial.

## Methods

### Study Design

We will use the RE-AIM implementation science framework [51] to guide the assessment of the program’s RE-AIM. We will pilot-test STARS using a type-1 effectiveness-implementation hybrid design in a racially and ethnically diverse sample of 60 EA-SGM individuals living in Philadelphia, PA, who report past-month suicidal ideation. Participants will be randomized to receive an in-person SPI (control arm) or to receive in-person SPI alongside STARS (intervention arm). After the baseline evaluation, we will follow participants for 6 months, evaluating them at 2, 4, and 6 months.

Consistent with our type-1 design, we will prioritize the preliminary effectiveness outcomes (suicidal ideation and behavior) and hypothesized mechanisms of change (coping with discrimination, social support, and positive affect). These measures will help us estimate critical parameters for a future trial as our *primary outcomes*. We will also monitor implementation targets related to reach, adoption, implementation, and maintenance as *secondary outcomes*.

This study is registered on ClinicalTrials.gov (NCT03678181).

### Participants and Enrollment Procedures

#### Eligibility Criteria

Participants will (1) be aged between 18 and 24 years, (2) live in the Philadelphia Metropolitan Area (given that we are most familiar with those emergency service options) and plan to live in the area for the next 6 months, (3) report past-month suicidal ideation, (4) identify as a sexual minority (ie, identify with a sexual identity other than heterosexual) or gender minority (ie, sex assigned at birth is different from gender identity), and (5) report owning and having regular access to a smartphone that they can use to participate in study activities. Those with symptoms of psychosis that are not adequately treated will be excluded.

#### Recruitment, Screening, and Enrollment

With increasing use of the internet, there exists a great opportunity for reaching traditionally hard-to-reach and vulnerable populations, including EA-SGM groups and racially diverse groups. We will reach the population using recruitment advertisements on social media platforms (eg, Facebook, Instagram, Reddit, and Twitter). We developed advertisements that promote our target population’s interest by including diverse images of youth (ie, images of different ages and portraying diverse races and ethnicities) and using advertisement-targeting specific to sociodemographic characteristics (eg, youth living in our region and who meet our age range) and interests (eg, youth following media with LGBTQ+ themes). Materials avoid identifying candidates as EA-SGM groups in the recruitment text to prevent unintended disclosure. Advertisements will link interested individuals to the study survey in REDCap (Vanderbilt University), where they may verify their eligibility, email the team, or locate a toll-free number if they want to learn about the study. We have recruited through these platforms for more than a decade with a significant track record of demonstrated success [55] and plan to use lessons learned from these efforts to guide the implementation of our proposed recruitment strategy.

We anticipate reaching our recruitment goal within 9 months of beginning enrollment. In previous trials in Philadelphia with sexual and gender minorities in this age group, we have found that >40% of individuals who clicked on our web advertisements through social media screen were eligible. Of those, 80% provided full contact information, and approximately 40% scheduled and attended an in-person enrollment visit. We expect that most (approximately 85%) will consent and enroll in the trial. Thus, for the proposed study, we expect to screen approximately 550 individuals, conduct full eligibility evaluations with approximately 70 (12.7%) EA-SGM individuals in person over the 9-month period, and obtain consent from and randomize 60 (10.9%) for our study. On the basis of these estimates, we will randomize 5 to 7 participants per month over the course of 9 months. As an enrollment strategy, we will provide complimentary rideshare to and from the baseline visit site as needed.

The web screener survey (through REDCap) will include initial consent to be screened, followed by questions that relate to eligibility based on our inclusion and exclusion criteria. Participants will be screened for self-reported psychosis using 2 questions taken from the Diagnostic Interview for Anxiety, Mood, and OCD and Related Neuropsychiatric Disorders screener [56]. Participants who report no active psychosis and meet all other criteria for eligibility will be asked the final eligibility question of whether they have had suicidal ideation in the past month. Participants who meet all the preliminary eligibility requirements will be asked to provide contact information and preferred mode of contact.

#### Phone Screening

Participants who are found to be preliminarily eligible via the web screening process will be contacted via phone. During phone screening, information about the potential participants’ physical location will be collected (if emergency services are needed) and a brief safety check will be conducted. Then, potential participants will be scheduled for their baseline evaluation.

#### Baseline Evaluation

If preliminarily eligible, participants will be scheduled for an in-person visit where they will complete the informed consent process and be evaluated by a licensed clinician for suicidal ideation and attempts using the Columbia–Suicide Severity Rating Scale (C-SSRS) [57]. Ineligible participants will be provided with referrals to mental health services based on their insurance. Enrolled participants will receive SPI, complete the baseline survey, and then be randomized. EA-SGM participants randomized to STARS will have immediate access to the app, be scheduled for their 6 peer mentor check-ins, and learn how they can reschedule future peer mentor sessions.

### Randomization

Enrolled participants will be randomized during the baseline visit through REDCap’s randomization module. A statistician created a block randomized list to ensure that there is an equal number of control and STARS participants, with a total of 102 assignments in blocks of size 4 or 6. Participants randomized to the control condition will complete follow-up assessments over 6 months. Although providing the gold-standard SPI to participants in the control condition may result in a floor effect, withholding this protocol would be unethical, given the participants’ vulnerability to suicide. This design will allow for a preliminary evaluation of whether the incorporation of STARS improves the use of SPI among EA-SGM groups.

### Control Arm

SPI [58] involves a collaborative and personalized conversation between the intervention facilitator and a person who is at high risk for suicide. SPI reduces the likelihood of future suicide attempts by collaboratively developing a written safety plan that consists of six steps (Table 1): (1) identifying warning signs, (2) implementing internal coping strategies, (3) using social interactions for distraction, (4) using one’s social network (family and friends) for help, (5) using professional resources for help, and (6) making the environment safe by reducing access to lethal means.

**Table 1:** Core components, scientific rationale, and measurement metrics.

Features	Description	Scientific rationale (measurement metrics)
Safety plan	Readable and editable digital version of the six-step Safety Planning Intervention, which includes: (1) warning signs, (2) internal coping strategies, (3) using social people and situations for distraction, (4) using one’s social network (family and friends) for help, (5) using professional resources for help, and (6) making the environment safe Phone numbers are tappable to facilitate calling Call 911 button	Increase the use of the Safety Planning Intervention [58] during times of crises by pairing the safety plan app content with peer mentor care navigator support (number of times accessed, number of times edited, and total time spent)
Resources and activities	Multimedia resources and information about relationships, support, identity, stress, emotional well-being, safety planning and suicide, discrimination, drugs and alcohol, and life skills—tailored for emerging adult sexual and gender minorities, inclusive learning styles and health literacy Robust management system to create new content and activities Activity types include quizzes, self-assessments, goal setting, choose-your-own-adventure, sorting (eg, sort activities into healthy or unhealthy coping), and matching formatted to promote skills acquisition (eg, match the emotion to a typical behavior)	Content corresponds to and aligns with the Integrated Behavior Model [50] (number of articles read and total time spent) Interactive activities to address suicide prevention and reinforce emerging adult sexual and gender minorities’ social support, coping with discrimination, and positive affect (number of activities completed and total time spent)
Peer mentor appointments	Schedule of upcoming peer mentor appointments with links to web-based meeting room List of completed peer mentor appointments‎	Provide social support, reduce the impact of discrimination, promote positive affect, and reduce barriers for emerging adult sexual and gender minorities to enact their Safety Plans (number of sessions attended)
Profile	Personalized username, customizable avatar, brief bio, badges earned, list of forum posts, and comments	Personalization, gamification, and cues to incentivize engagement (number of times visited)
Goals	Select from researcher-created goals or create new goals with milestones, tasks, and a journal feature	Goals feature builds on self-management skills (number of goals set and milestones and tasks completed)
Forum	Start or add to existing discussions; upload images, videos, memes; and so on Favorite posts and follow others Staff monitor and add to forum posts and include polls to encourage dialogue	Peer support for resilience and behavior change (number of posts, comments, and likes; total time; and content of posts)
Gamification	Sophisticated tracking of app use to trigger behavior-specific rewards Badges awarded for tracked events	Gamification [59] features incentivize continued engagement (number of log-ins and badges earned)

### Intervention Arm

In the STARS intervention, our transdisciplinary team will develop and pilot-test an intervention to reduce suicidal ideation and behaviors among EA-SGM groups. STARS will include content about suicide prevention, including safety planning and targeting positive affect, and discrimination and social support. STARS will consider the unique cultural factors that may influence the efficacy of safety planning for EA-SGM groups (eg, access to LGBTQ+ resources) and include supports that promote the use of a suicide safety plan constructed with a licensed clinician.

### Theoretical Foundation

**Figure 1:**
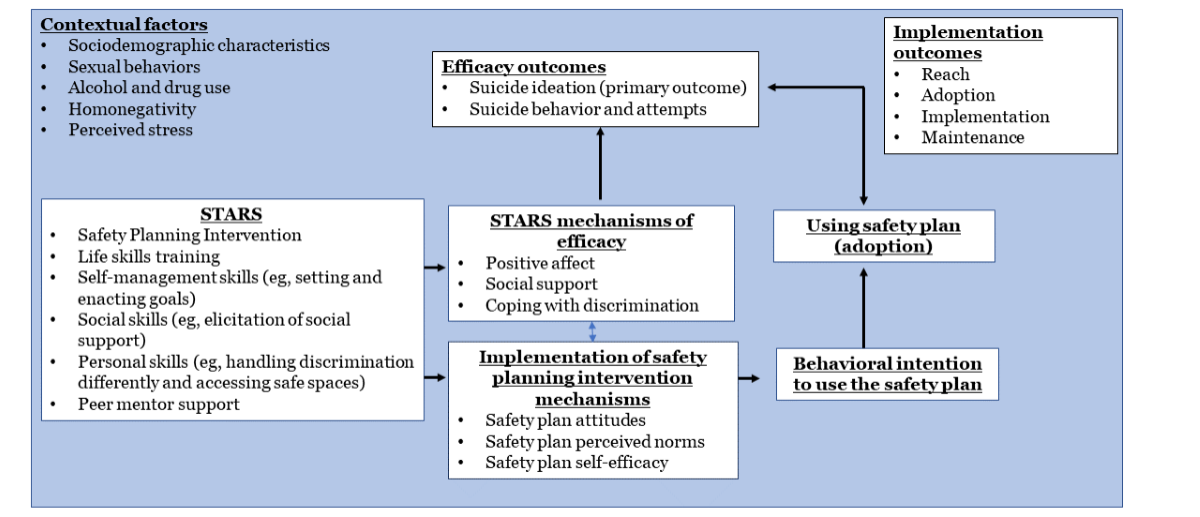
Conceptual model. STARS: Supporting Transitions to Adulthood and Reducing Suicide.

The Integrated Behavior Model informs STARS in that the intervention acknowledges that EA-SGM groups’ attitudes, norms, self-efficacy, and intentions are key constructs to target to effect behavior change (Figure 1). For STARS, we will adapt the Integrated Behavior Model–informed intervention messaging to promote EA-SGM groups’ use of the safety plan during times of crisis by improving participants’ attitudes toward, intention to use, and self-efficacy in using their safety plan (Multimedia Appendix 1). By enlisting peer mentor support, STARS will also target perceived norms about the frequency of safety plan use among their peer group. In addition, STARS will include a copy of the participant’s safety plan and have topics to facilitate using the plan, with special attention dedicated to differences in social networks and previous negative experiences with health care to improve the impact of SPI among EA-SGM groups [11,60]. STARS provides life skills training (eg, building affirming relationships, applying to schools, study skills, resume-writing skills, and emotion management skills; Table 1) and peer-delivered motivational interviewing (to support the use of the safety plan), through which, EA-SGM groups will learn about suicide prevention, set goals to shift their attitudes toward their safety plan, increase their intention to use their safety plan, and engage with a community that understands their needs and norms. This will help participants to manage their suicidal thoughts without engaging in suicidal behavior.

Consistent with the Integrated Behavioral Model, we recognize the importance of bolstering the linkage between intention and behavior to use their safety plan. To support this linkage, STARS will focus on three major skill sets: (1) self-management skills (eg, setting and enacting goals to promote positive affect), (2) social skills (eg, elicitation of social support), and (3) personal skills (eg, coping with discrimination more effectively and seeking safe spaces). With the support of peer mentors, EA-SGM groups can set goals to shift their sense of mastery (to improve positive affect), engage with a community that understands their needs (to improve social connection), and identify local resources that support their well-being (to improve coping and reduce the impact of discrimination on their well-being). Finally, STARS will promote the use of the safety plan by equipping peer mentors to discuss barriers to enacting the safety plan.

STARS will address theoretical mechanisms that can reduce the severity of suicidal ideation, including coping with discrimination and improving social support. STARS additions will include a module about promoting positive affect through behavioral activation, pleasant events scheduling, and safety plan content. STARS will address constructs of self-efficacy, knowledge, and attitudes about safety planning.

### Intervention Components

STARS uses traditional multitier architecture in which the concerns are separated into presentation, service, and database *layers.* The administrative portal was written in React (Meta Platforms), whereas the mobile app is written in React Native, which allows us to build native apps for both Android and iPhone, reusing approximately 90% of the code between the 2 operating systems. Table 1 presents brief descriptions of the core components of STARS, aligned with the theoretical underpinnings for addressing stigma and changing behavior.

The adaptation of the existing iREACH content was guided by ADAPT-ITT (Table 2). The ADAPT-ITT model was developed to adapt evidence-based practices for circumstances or populations outside those included in the evidence base. The ADAPT-ITT model includes 8 phases. These include assessment, decision, adaptation, production, topical experts, integration, training, and testing. This framework is useful because implementation of evidence-based practices often requires modification to the innovation [53]. Following a framework such as ADAPT-ITT can speed the process of adaptation, while incorporating feedback from peer mentors and a technical expertise group (TEG) with expertise in LGBTQ+ health, youth development, and suicide prevention.

From September 2021 to September 2022, the team met weekly to review the existing content and features on the platform to identify the required modifications, to ensure readiness for the implementation of the pilot test (*assessment*). During the *decision* stage, we reviewed evidence-based practices and identified the components that were ready for adoption and those that needed to be adapted. For example, although SPI did not require adaptation, the research team built SPI into the app for STARS. We revised the iREACH content and interactive activities to address suicide prevention and reinforce EA-SGM groups’ social support, coping with discrimination, and positive affect. Our review included both text and imagery and video content within the platform to ensure developmentally appropriate, contextually relevant, and culturally sensitive alignment with EA-SGM groups. Over the following months, the research team met weekly to discuss the features, design, and engagement desired within STARS *(administration)*. During these discussions, we reflected about whether the platform met the desired expectations for design and ease of use and incorporated feedback regarding its content and layout accordingly (Multimedia Appendices 2-6).

**Table 2:** Modifications to the intervention content using the assessment, decision, administration, production, topical experts–integration, training, and testing framework (8 steps).

Phase	Procedures
Assessment (months 1-8)	Obtain iterative feedback about content adaptation from the research team and technical expertise group
Decision (months 2-8)	Review the information obtained from the assessment and draft the revised intervention content (text, video, and activities) and measures
Administration (month 9)	Elicit feedback through iterative testing and feedback cycles Address reactions and make modifications
Production (months 2-12)	Balance priorities while maintaining fidelity to core elements and underlying theoretical framework
Topical experts (month 10)	Elicit expert review of the final curriculum and activities
Integration (months 11-12)	Integrate content from experts and conduct readability tests Translate curricula into a mobile platform
Training (months 11-12)	Beta test the study procedures, recruit peer mentors, and train the staff Begin recruitment for pilot testing
Testing (months 18-36)	Pilot-test the intervention with 60 emerging adult sexual and gender minority individuals Collect implementation and preliminary efficacy data Analyze the data

During the *production* phase, our technical partner produced instances of the app based on the elicited feedback and began to iteratively test for platform stability, identification of bugs requiring modification, and ability to track engagement metrics. The test environment looked as similar to production as possible, so that changes and bug fixes could be tested and reliably expected to work when promoted to the production environment. The research team used the beta versions of STARS for internal usability testing. We worked with our technology partner to finalize the app and ensure its security using industry best practices.

Our TEG served as *topical experts,* offering their subject matter expertise on the content and activities incorporated into the app. After the TEG provided written comments, we integrated their comments and tested the readability of the finalized content using the Flesch-Kincaid Readability Test (average score 6.98, SD 1.8) [61].

During the *integration* stage, the research team worked with the technical partner to make the final refinements and modifications before launching the production server. We reviewed the production server instance to ensure that the layout works with different mobile devices and browser platforms as intended and that all data are captured and saved in the server as planned.

During the *training* stage, the research team worked on reviewing the study-related procedures and finalizing the protocols’ operations manual. The recruiters were trained to fidelity in the process of describing the study to EA-SGM individuals who call to inquire about participation and suicide risk assessment as potential participants will indicate previous suicidal ideation in the past month. Research team members were trained in data management, which includes securely storing data, data protection, and proper data collection. All staff were trained in accordance with National Institutes of Health standards on recognizing and reporting adverse events.

The research team also finalized the peer mentor modules and began the recruitment and training of peer mentors using the existing training and fidelity protocols (Multimedia Appendix 1). STARS relies on peer mentors as care navigators to provide social support, reduce the impact of discrimination, connect youth to safe spaces, remind them to use their safety plan at the beginning and end of every session, and brainstorm and implement solutions to overcome obstacles to its use. For example, at each session, the peer mentor will check in on whether the participant used their safety plan and inquire about how it went, about barriers to its use or efficacy, and about whether any changes are needed to the safety plan. This approach offers a confidential and user-controlled opportunity to support EA-SGM groups in their transition to adulthood.

Peer mentor positions were posted on the web; applicants were asked to provide their curriculum vitae and a 1-paragraph description of any previous experiences with or interest in working with LGBTQ+ emerging adults (aged 18-24 years). This allowed for the selection of candidates who would be suitable as peers for the project. Once selected, peer mentors completed 12 hours of training with a licensed clinical psychologist, which included didactics, modeling, role-plays with feedback, and discussions about difficult topics that might emerge outside the planned content. Following training, they attended weekly group supervision for 1 hour. Peer mentors were trained to conduct 6 sessions (which were audio recorded) with participants to provide social support, reduce the impact of discrimination, promote positive affect, and reduce barriers to enacting the safety plans (refer to the peer mentor manual in Multimedia Appendix 1 for additional details). Staff identified measures of fidelity specific to each peer mentoring session that were assessed for each audio-recorded session. Peer mentors were paid on an hourly basis.

The peer mentors do not conduct SPI, but they help participants brainstorm ways to overcome obstacles to using SPI. Peer mentors receive booster trainings every 2 months to reinforce their skills and respond to emerging challenges [62]. Peer mentors’ proficiency is monitored via audio-recorded role-plays using standard coding protocols. Peer mentor sessions occur on a Health Insurance Portability and Accountability Act–compliant telehealth platform.

### Testing

The final ADAPT-ITT phase evaluates the intervention in a pilot trial described in the following section. A randomized clinical trial (N=60) is recruiting individuals aged 18 to 24 years who identify as sexual or gender minorities and who report past-month suicidal ideation. After completion of an interest survey and phone screening, participants complete a comprehensive eligibility assessment in person with a licensed clinician. All eligible participants complete SPI in an original, unadapted format. Then, participants are randomized to either the STARS or control group. The control group completes assessments, which include a survey and an interview, every 2 months (2-, 4-, and 6-month follow-up). Participants in STARS meet with a research assistant to download and complete an orientation to the STARS app and to schedule the peer mentoring sessions, conducted virtually. The STARS participants then complete the same assessment as the control group (at 2, 4, and 6 months). User data are collected from the STARS app, as is feedback from the peer mentors about the way that time was spent in their sessions.

### Sample Size and Power Analysis

We plan to recruit 60 EA-SGM participants. A power analysis in Repeated Measures with Attrition: Sample Sizes for 2 Groups (5% attrition at each time point; 2-sided test; *r*=0.50 between tests; Cronbach α=.05; power=0.80) suggested that we would need 29 participants per group to detect a medium between-group effect size on suicidal ideation (Cohen *d=*0.60). Previous studies found that crisis response planning (a type of safety planning) had a between-group effect size ranging from 0.0 to 0.7 (at the 1-, 3-, and 6-month follow-up) compared with contracting for safety [39]. We anticipate that with the addition of important skills (coping with discrimination, social support, and positive affect) and peer support, a medium effect size improvement in suicidal ideation over safety planning alone is realistic and clinically meaningful.

There is controversy regarding the use of “pilot data” to estimate actual effect sizes [63]. The primary goal of this project is to determine our intervention’s reach, feasibility, acceptability, and its implementation fidelity. Our secondary goal is to ensure that the critical parameters required to plan efficacy targets within a large trial are estimated. We seek to estimate key study parameters with sample means and proportions together with 2-sided 95% CIs and test the primary null hypotheses at the traditional 2-sided level α=.05. These data can help rule out unusually large or small effects by confirming that extrinsic effect sizes are contained within our CIs and help inform a future efficacy trial.

### Engagement and Retention

#### Retention

We will make all efforts to retain consented participants. Our study retention plan draws on previously successful retention protocols to achieve at least an 80% retention rate. It is critical to obtain accurate follow-up contact information. We will use best practices to retain participants (eg, comprehensive locator information that includes participants’ mobile phone number, email, Facebook and Twitter username, and contact information about 2 peers who could help us contact them), while being sensitive to undue disclosure of EA-SGM individuals participating in the study.

We also have a planned schedule of follow-up that consists of a variety of follow-up methods. Short time (2 months) between assessments will help us quickly address retention issues. A study-specific home page allows study staff to monitor participant progress at a glance, viewing steps or phases in the dashboard filtered by conditions such as record status, intervention arm, last log-in to the app, and so on. This dashboard facilitates participant retention via prompts to study staff to reach out to participants who have missed a specified number of study activities and by providing a secure platform to communicate with staff and deliver participant incentives. At each follow-up assessment, participants can update their contact information. This allows us to remain connected with participants and facilitates the delivery of reminders regarding upcoming study procedures.

Initially, a participant who does not respond to an electronic notification that a survey is due will automatically receive additional notifications 36 hours after the initial notification. If the participant has still not completed the assessment 24 hours after the third electronic notification, a research staff member will escalate contact intensity. Depending on the participant’s preferences provided upon registration, they will be contacted initially through the preferred mode of recontact (eg, via SMS text message); if still unresponsive, other available modes (eg, phone call) will be used. Each contact is logged in an electronic retention system.

#### Incentives

Participants will be followed for 6 months after enrollment. We have designed our incentive schedule to encourage the completion of study assessments, in that after the baseline assessment, which is in person and for which participants are compensated US $50, each of the subsequent assessments pays the participant at an increasing rate to encourage them to complete future assessments. They will be compensated US $30 for completing the 2-month assessment, US $40 for the 4-month assessment, and US $50 for the 6-month assessment. This incremental compensation has proven highly effective in our previous cohorts. Neither our institutional review board (IRB) nor previous advisory boards have found this amount of incentive to be coercive. Rather, they have found it commensurate with what would be expected to compensate participants if they were engaged in face-to-face and web assessments that are similar in duration.

### Outcomes

#### Overview

Assessments will be conducted on the web using REDCap (Table 3). All participants will complete web surveys every 2 months (2-, 4-, and 6-month follow-up surveys), which are similar to the baseline survey they completed in person.

**Table 3:** Measures for primary and secondary outcomes.

Outcome			Measures
**Recruitment**			
	Recruitment and enrollment (Reach)	Advertisement and screening metrics	
**Primary outcomes**			
	Suicidal ideation (efficacy)	Columbia–Suicide Severity Rating Scale; Severity of Suicidal Ideation Subscale [57] (primary outcome); Intensity of Ideation Subscale (secondary outcome); Suicidal Behavior Subscale (secondary outcome); at baseline, we will assess lifetime and past-month suicidal thoughts and behaviors; and at follow-up assessments, we will assess suicidal thoughts and behaviors in the past 2 months	
**Secondary outcomes**			
	Safety Planning Intervention and STARS^a^ app willingness (adoption)	Self-report of willingness to use and satisfaction with the Safety Planning Intervention and the STARS app and Short Intervention Evaluation Form [64]	
	Safety plan and STARS app use (participant implementation)	Self-reported use of the safety plan, STARS app log-in sessions, app session length, pages visited, and activities completed	
	STARS fidelity (peer mentor implementation)	Peer mentor session adherence and competence	
	Retention (maintenance)	Completion of evaluations	
	Expert and peer mentor perceptions (maintenance)	Qualitative interviews with the youth action group and technical expertise group	
**Mechanisms of action**			
	Social support and isolation	5-Item Scale of Parental and Peer Support [65], Interpersonal Needs Questionnaire–Reduced [66], and UCLA Loneliness Scale [67]	
	Positive affect	PANAS^b^-positive [68,69]	
	IBM^c^ targets	Attitudes, norms, self-efficacy, and intentions to use safety planning [70]	
	Coping with discrimination	Coping with Discrimination Scale [71,72] and sexuality-related stressors [73]	
**Covariates**			
	Sociodemographics	Demographics and technology use [74]	
	Sexual behavior	Sexual Practices Assessment Schedule [21,75]	
	Alcohol and drugs	Alcohol Use Disorder Identification Test [76] and Alcohol, Smoking, and Substance Involvement Screening Test [77]	
	Homonegativity	Internalized homonegativity scale [78]	
	Gender-related stigma	Gender minority stress and resilience rejection subscale [79]	
	Perceived stress	Daily hassles over the past month [80]	
	Discrimination	Everyday Discrimination Scale [81,82]	

^a^STARS: Supporting Transitions to Adulthood and Reducing Suicide.

^b^PANAS: Positive and Negative Affect Schedule.

^c^IBM: Integrated Behavioral Model.

#### Primary Outcomes

The RE-AIM framework guides our selection of efficacy and implementation indicators. Consistent with the type-1 hybrid design, the primary goal of our pilot study is to identify key indicators of preliminary efficacy, with a secondary goal of describing the implementation context.

#### Reach

We will track advertisement impressions (clicks, cost/click, and click-through rate), screening (number of participants who were screened, were eligible, and provided contact information and cost/eligible contact), and enrollment metrics (number of eligible participants who attend in-person visits and enroll). We will document the reasons for refusal among eligible individuals. We will compare reach across social media platforms.

#### Efficacy

The C-SSRS is a clinician-rated interview measure of the severity of suicidal ideation (primary outcome), intensity of ideation (secondary outcome), and suicidal behavior (secondary outcome) with strong psychometric properties that will be our primary efficacy indicators [57].

#### Adoption

We will evaluate changes in the intention to use the safety plan from baseline through all follow-up assessments. We also will examine satisfaction with and perceived usefulness of the components in their assigned arm. Participants will also complete the Short Intervention Evaluation Form [64] to elicit information about their experience (ie, was the intervention interesting, was it relevant, or did they learn from the intervention). For STARS participants, we will evaluate self-reported intention to use the STARS app and satisfaction with the app.

#### Implementation

To assess intervention engagement by *participants*, we will measure counts of log-in sessions to the app, app session length, pages visited, activities completed, and other functions used within the app, which can indicate intervention dosage. We will also collect the number, frequency, and duration of peer mentor sessions that participants requested and attended and assess self-reported frequency of use of the safety plan in both conditions. *Peer mentors* will complete a case report form after each session to document the topics discussed. Procedures for monitoring intervention adherence and competence include audio recording all sessions and coding for clinical skill or competence. We will interview the *youth advisory group, TEG, and peer mentor members* for their reflections about lessons learned, insights into potential modifications, and experienced challenges and opportunities gleaned during implementation. Semistructured interviews will ask about the intervention adaptation process and perceived impact of the intervention. Interviews will last 30 minutes, will be audio recorded, and transcribed. We will create a codebook of a priori and emergent themes including operational definitions of all codes and sample quotations. Then, 2 study team members will independently code the data; a third team member will review the coded sections and resolve discrepancies.

#### Maintenance

**Figure 2:**
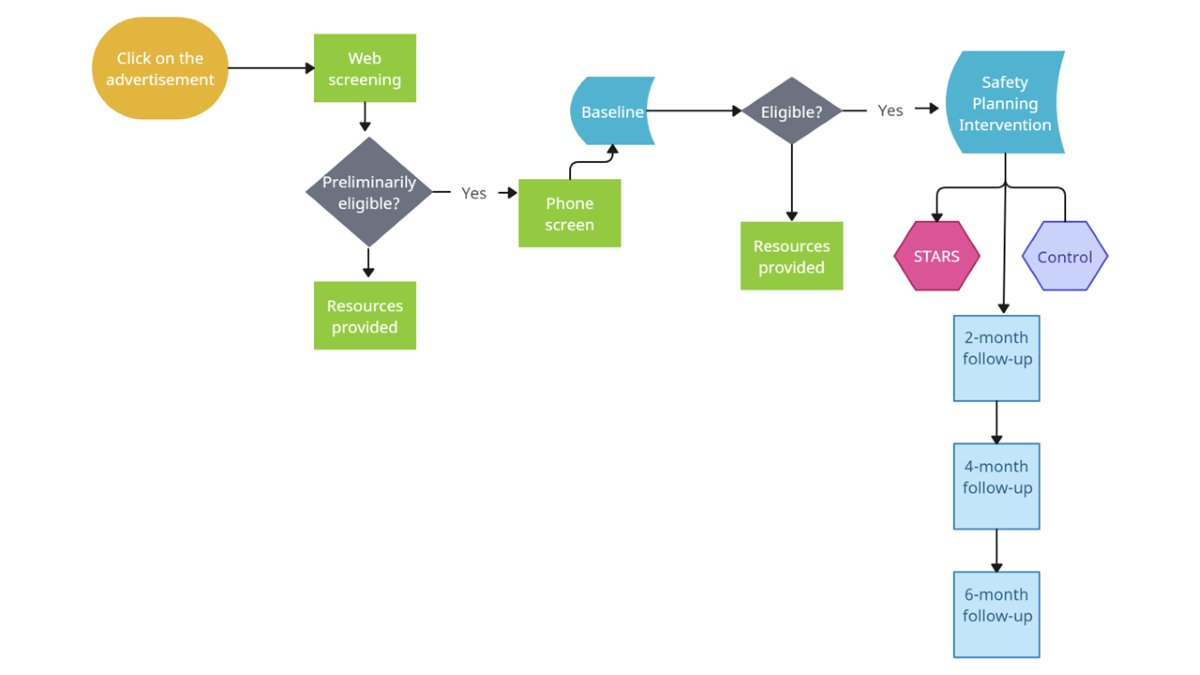
Timeline for participation. STARS: Supporting Transitions to Adulthood and Reducing Suicide.

We will monitor retention over time as a marker of maintenance, with a criterion of ≥80% retention at the 6-month follow-up (refer to Figure 2 for the timeline). We will compare those who are lost to follow-up with study completers across baseline scores based on our key efficacy indicators and sociodemographic characteristics.

#### Mediators of Intervention Effects

We will also measure mechanisms of action (Integrative Behavioral Model targets [70], social support, positive affect, and coping with discrimination) and key demographic and behavioral covariates.

##### Social Support

Social support from parents and friends will be measured separately using a 5-item emotional support scale, with items rated on a 5-point scale. The measure has strong convergent and divergent validity, test-retest reliability, and internal consistency. Participants will complete the UCLA Loneliness Scale [67], a 20-item measure of subjective experiences of loneliness, with items rated on a 4-point scale, reflecting the frequency with which a participant feels a particular way (often, sometimes, rarely, or never). The measure has strong convergent and divergent validity, test-retest reliability, and internal consistency. The Interpersonal Needs Questionnaire–Reduced [66] is a 15-item measure of perceived burdensomeness and thwarted belongingness. The measure has strong psychometric properties, including measurement invariance, divergent and convergent validity, and ability to predict future suicidal ideation.

##### Positive Affect

The Positive and Negative Affect Schedule–Positive Affect subscale [68] is a 10-item measure of positive affect over the past week, including enthusiasm, interest, determination, excitement, inspiration, alertness, activity, strength, pride, and attentiveness. Each item is rated on a Likert scale ranging from 1 (very slightly or not at all) to 5 (extremely). The Positive and Negative Affect Schedule has strong internal consistency and convergent and divergent validity.

##### Integrative Behavioral Model Questionnaire for Safety Planning

We will measure attitudes, norms, self-efficacy, and intentions regarding adoption of the safety plan using an adapted version of the Theory of Planned Behavior Questionnaire [83]. The measure has strong psychometric properties, including high reliability and validity.

##### Coping With Discrimination

The Coping with Discrimination Scale [71] is a 41-item measure assessing internalization (10 items), disengagement (5 items), drug and alcohol use (5 items), support seeking (5 items), resistance (6 items), resilience (6 items), and education or advocacy (8 items). Participants respond to these items using 6 response options, ranging from 1 (never like me) to 6 (always like me). Instructions for the measure are as follows: “This is a list of strategies that some people use to deal with their experiences of discrimination. Please respond to the following items as honestly as possible to reflect how much each strategy best describes the ways you cope with discrimination. There are no right or wrong answers.”

#### Covariates

We will measure the following constructs as covariates: sociodemographics (age, race and ethnicity, education, employment, income, sexual orientation, sex assigned at birth, and gender identity), sexual behavior, alcohol and drug use, internalized homonegativity, experiences of everyday discrimination, and perceived stress. These measures are presented in Table 3.

### Statistical Analysis

Before conducting our multivariate analyses, we will examine the study variables using descriptive statistics and test for differences across demographic characteristics (eg, race and ethnicity, age, and education) using 2-tailed *t* tests, ANOVAs, and chi-square tests, as appropriate. As participants will be randomized to the 2 treatment groups, systematic baseline differences are not expected; however, if some parameters differ across conditions at baseline, they will be included as covariates in subsequent multivariate models. We will calculate descriptive summary statistics corresponding to the study variables at each visit to understand any temporal patterns and compare the 2 treatment groups in terms of average change from baseline after intervention (averaged across all 3 follow-up times).

In its simplest form, under the assumption of equivalence between the 2 randomized groups, we will compare differences from baseline to the 6-month outcomes across our outcome measures using 2-tailed *t* tests, ANOVAs, and chi-square tests, as appropriate. For repeated measures analyses, we will use the general framework of generalized linear mixed models to model the longitudinal outcomes. We will compare differences across the groups over time based on the C-SSRS suicidal ideation subscale, social support (a composite measure of the 5-Item Scale of Parental and Peer Support, Interpersonal Needs Questionnaire, and University of California, Los Angeles Loneliness Scale), coping with discrimination (a composite measure of Coping with Discrimination Scale and a measure of sexuality-related stressors), and positive affect (Positive and Negative Affect Schedule–Positive), noting that we will not be powered to detect differences in suicidal behavior; however, we will have sufficient power to detect changes in the severity of suicidal ideation. We will evaluate the preliminary evidence for associations between change in intervention targets, mechanisms, and suicidal ideation or behavior. We will examine differences in the rate of use of the safety plan and in retention by condition, and we will examine whether paradata from STARS (ie, frequency of peer mentoring sessions and using the app) correlate with safety plan use.

### Missing Data

We will compare those who are lost to follow-up with study completers across baseline scores based on our key efficacy indicators (C-SSRS suicidal ideation and suicidal behavior subscales, social support, coping with discrimination, and positive affect) and on sociodemographic characteristics using linear regression. Web-based entry for all measures will help reduce missing data. We will manage missing data by applying likelihood-based methods that include partial information about participants who drop out prematurely and develop nonresponse adjustment and poststratification weights to account for survey nonparticipation. The use of expectation-maximization algorithm and multiple imputation approach in longitudinal analyses will help overcome missing data concerns when appropriate. When the missing-at-random assumption is not supported, we will not replace missing data to avoid bias.

### Ethics Approval

The human participants’ considerations presented in this protocol are approved by the IRB of the University of Pennsylvania (protocol number 849500).

## Results

This project began in September 2021. Preliminary study activities (eg, obtaining IRB approvals, hiring and training staff, and developing the study protocol and study-specific procedures) were completed in September 2022. During this period, we also worked with our technology partner (One Cow Standing), our youth advisory group, and a TEG to update and expand the intervention content related to suicide ideation; finalized and programmed all study tools; and established robust recruitment, retention, and engagement plans. Recruitment began in October 2022; we anticipate completing recruitment by September 2023. Final participant follow-up will be completed by February 2024, followed by data analysis. Study results will be disseminated in 2024.

## Discussion

### Summary

The proposed project is innovative in 3 ways. First, although evidence-based life skills programs target diverse racial and ethnic and urban or rural youth [84,85], to the best of our knowledge, ours will be the first life skills intervention designed to reduce suicide risk among a diverse sample of EA-SGM groups who are at risk for suicide by promoting positive affect, coping with discrimination, and improving social support. Although a study found that interventions targeting positive affect reduced suicidal ideation to a greater extent than those targeting negative affect [86], this has not been demonstrated among EA-SGM groups. Second, we will be among the first to tailor and study the effects of an e-delivered life skills intervention to reduce EA-SGM groups’ suicidal ideation and behavior among sexual minorities. The life skills app will deliver tailored and interactive content and reinforce it with peer mentor support. Third, our intervention acknowledges that EA-SGM groups might require ongoing support in enacting their safety plan and leverages our previous success in teaching motivational interviewing to young lay workers to facilitate care navigation and support of vulnerable youth. The peer mentors’ ongoing support in problem-solving obstacles to the use of the safety plan and reminding EA-SGM individuals to use their safety plan will serve as key implementation strategies to enhance the impact of this intervention.

### Principal Contributions to the Field

In light of the difficulty in using “pilot data” to estimate effect sizes [63], the primary goal of this project is to determine STARS’ reach, acceptability, and implementation fidelity. Our secondary goal is to estimate the critical parameters required to plan efficacy targets within a large trial. Although we anticipate having adequate power to determine the effects on suicidal ideation, we will not be powered to determine the effects on suicidal behavior, which will be the goal of a fully powered future R01. These data can help rule out unusually large or small effects by confirming that extrinsic effect sizes are contained within our CIs [63] and help inform a large-scale version of the intervention to test its efficacy in reducing suicidal ideation and behavior among EA-SGM groups.

## Appendix

Multimedia Appendix 1 Peer mentor manual.

Multimedia Appendix 2 Forums.

Multimedia Appendix 3 Goal setting.

Multimedia Appendix 4 Safety plan.

Multimedia Appendix 5 Articles and activities.

Multimedia Appendix 6 Peer mentor scheduling.

## Abbreviations

ADAPT-ITT: assessment, decision, administration, production, topical experts–integration, training, and testing
C-SSRS: Columbia–Suicide Severity Rating Scale
EA-SGM: emerging adult sexual and gender minority
IRB: institutional review board
LGBTQ+: lesbian, gay, bisexual, transgender, queer, plus
RE-AIM: reach, efficacy, adoption, implementation, and maintenance
SPI: Safety Planning Intervention
STARS: Supporting Transitions to Adulthood and Reducing Suicide
TEG: technical expertise group

## Data Availability

Data will be deposited in the National Institutes of Health data repository.
