# Supplementary material for: A Suicide Prevention Intervention for Emerging Adult Sexual and Gender Minority Groups: Protocol for a Pilot Hybrid Effectiveness Randomized Controlled Trial
Source: JMIR Res Protoc. 2023 Sep 29;12:e48177. doi: 10.2196/48177 (PMC10576233; doi:10.2196/48177)
Supplement: Multimedia Appendix 1 [file resprot_v12i1e48177_app1.pdf]

Session 1: Rapport building, Safety Planning, and Orientation to STARS App (24 min)

Session 2: Building a life worth living

Session 3: Dealing with negative self-talk

Session 4: Working to build positive experiences in your life

Session 5: Dealing with people who are hurting you

Session 6: Managing relationships

Session 1: Rapport building, Safety Planning, and Orientation to STARS App (24 min)

1) Introductions (5 min)
2) Agenda Setting (1 min)
3) Orientation to Peer Mentor Sessions (3 min)
4) Safety Planning (10 min)
5) Orientation to STARS Apps (5 min)

Introductions (5 min)

- Exchange names (and pronouns if desired)
    - Hi there. My name is James. I use he/him pronouns. And I will be the Peer Mentor working with you today. Welcome! How should I refer to you as we meet today?

- Share a bit about what drew you to work as a peer mentor (sharing only what you feel comfortable sharing and supports rapport-building).

  For example: *"I am glad to get to chat with you today. As someone who identifies as gay, I know how much being able to connect with other LGBTQ+ people has meant to me. My hope is to be able to offer that opportunity here as we talk through some of the topics on which the STARS app focuses. But my other hope is for this to be a collaborative time, to decide together how best to spend our time. The overall goal of our time together is to collaboratively brainstorm and implement ways to help you build a life that you feel is worth living, and one that reduces suicidal thoughts."*

- Cover housekeeping items

  For Example: *"Before we get too far, I do need to cover just a few housekeeping things if that is okay with you."*
    - Sound check
    - Review of privacy and confidentiality

      For example: "*As a reminder, since this is a research study, we will be keeping some records about these sessions. We will keep a voice recording of our conversation to help with our analysis. I will also take some notes to make sure that I remember what we discussed. What we talk about today will remain confidential, meaning I will not share it with anyone outside of the research team. The only exception to the confidentiality rule is if I become concerned about your immediate safety or the immediate safety of someone else, in which case I need to alert someone on the research team so that they can make sure everyone is safe. Do you have any questions for me about that?"*

    - Ask the participant if there is anything you can do to make them feel more comfortable with this process.

      For example: "*I'd like to hear a bit more about you, but before I do, is there anything I can do that would help you to feel more welcome and at home during our call today? Or any questions that you have for me before we move on?"*

Agenda Setting (1 min)

- -For example: "*Now that we know a bit about each other, let's talk about how to spend our time on this call. I have a few suggestions based on how we usually spend the first STARS peer mentor session, but would love your input as well. Usually, we begin by talking about the goals and structure of these peer mentor sessions. Then, we talk some about the Safety Plan you created with Dr. Brown at your initial study visit. We can take a look at the STARS app together and make sure you know where to find things. And then we typically discuss plans for future sessions and wrap up. How does that sound to you? Anything you'd like to add to the agenda?"*

Orientation to Peer Mentor Sessions (3 min)

-Describe the goals of the peer mentor sessions

-For example: "*The goals of these peer mentor sessions are to provide an opportunity for us to collaboratively talk through some of what the STARS app has to offer. My goal is to guide us in a conversation in which we strategize together how to make the app content and tools useful to you. Solutions only work if they fit your circumstances, needs, and vision for your life So, we might brainstorm ways you want to use STARS, as well as to discuss the real life feedback you can provide on what works and what doesn't. Typically, each week we'll also check in on your Safety Plan, how you use it, and any challenges to using it or ways we can make it more helpful. We'll plan to do some of that today too. How does that sound to you?"*

-Describe the structure of the peer mentor sessions

-For example: "*These sessions will usually last between 20 and 30 minutes. If you need to reschedule a session, you can contact the peer mentor team using the Messages feature in the app menu. It's no big deal to reschedule a session or two, as I know things come up. We will meet 6 times for these sessions, and the goal is to meet once per week. Any questions about that?"*

Safety Planning (10 min)

-Pulling up the Safety Plan on the app

-For example: "*Okay, let's dive in to discussing the Safety Plan. I know that when you met with Dr. Brown, you made an individualized Safety Plan. I wanted to first remind you where to find your plan on the app—you can pull it up by clicking the "safety plan" icon at the bottom of the STARS home screen.*

-Asking whether they have used it since it was generated

-For example: "*Now since you met with Dr. Brown on [enter date, likely last week], have you tried to use your Safety Plan at all?"*

-If no: "*What got in the way of using it? Did you not have any thoughts about suicide this week?"*

-Consider jumping ahead to barriers section below.

-If yes: "*I'm glad that you already tried using it. How did it go?"*

-If good, provide reflection and affirmation. For example: "*You noticed that your urges for suicide were starting to rise and reached out to your friend, XXXXXXX, for help before it got worse."'*

-If bad: *"I'm sorry to hear that it wasn't helpful. What do you think prevented it from being helpful to you in that moment?"*

-If edits are indicated, jump ahead to edits section below.

-If buy-in boosts are needed, jump ahead to buy-in section below.

-Buy-in boosting (if needed)

-For example: *"You considered using the STARS Safety Plan but then had the thought "Safety plans aren't helpful" and decided not to use it. It sounds like in order for the STARS Safety Plan to be something you would use, it would have to feel more helpful. Perhaps we could talk some about what would make the STARS safety plan feel more useful to you.*

Remember that research shows that suicidal crises come and go. People who have had one in the past are very likely to have one in the future. Research also shows that when we're in a suicidal crisis, our brains are not as good at problem solving as they usually are. It's hard to plan, for instance. That's why it's so helpful to have a plan in advance—it takes the effort out of the equation so that you don't have to think about what to do. All you have to do is click the Safety Plan icon on the STARS home screen and you'll have a list of strategies waiting for you to use. You don't always have to start at the beginning, but you can if that's useful."

-Inquiring about whether any edits should be made to it

-For example: "*As you're looking at the Safety Plan now, does anything strike you as something that you know should change? Is it alright with you if we go ahead and update that now? How should that look instead?"*

-Asking about what might make it easier to use (Facilitators)

-For example: "*What would make it easier to use the Safety Plan?"*

-Asking about what might make it harder to use (barriers)

-For example: "*What would make it harder to use the Safety Plan?"*

-Reminder that anytime you're in a suicidal crisis, the goal is to pull up the Safety Plan and use it (the goal is to take the "mental work" out of it)

-For example: "*Remember that the goal is to pull the Safety Plan up when you're in a crisis so that it takes the energy out of planning how to get through the crisis."*

-Solicit questions/points for clarification about the Safety Plan

-For example: "*Any further changes or questions about the Safety Plan?*"

Orientation to STARS Apps (5 min)

-For example: "*Over the next 5 sessions, the plan is for us to spend some time with key topics from the STARS app. You don't have to wait for me to walk you through this to use the features—feel free to use anything that you think would be helpful. I'll point out a few places you could consider starting your explanation:*

- *The home screen has a menu at the bottom which connects you to some of the main sections of the app:*
  - *The Explore section offers some LGBTQ+ takes on navigating parts of life like relationships, emotional well-being, and connecting with supportive community.*
  - *We have already talked about the Safety Plan, which you can find on this menu to view or update whenever you want to.*
  - *The Appointments icon will show you your upcoming Peer Mentor sessions. You can also contact us about schedule changes on that page.*
  - *There is a Forum as well, where you can interact with other STARS community members about STARS content, posting your take, asking questions to the community, or browsing what other STARS users are thinking about.*
- *There are other features to find too, like your app profile where you can customize your own avatar, badges you can earn by using the app, and a place where you can set and track your goals.*

Do you have any questions about the app's features or how to use them?"

Feedback

-For example: "*It was great to meet with you today. As a way to help us develop a good working partnership, I'd love to get your feedback on how this meeting went and anything that I can do to make future sessions more helpful. How was our time together for you? Were there any things about today's discussion that were unhelpful or irritating or upsetting to you? Anything I can do differently in the future? Anything that was helpful? Keep me posted as we go along so that I can adjust to meet your needs.*"

Summarize and close

- Close with brief summary, particularly highlighting any of the following that occurred
  - Any generated motivations to use the safety plan
  - Acknowledging changes made to safety plan and express interest in feedback as they try out the changes
  - Any substantive feedback they provided about the session
- Mention next week's topic (Values and Goals)
- Thanks and closing

Session 2: Building a life worth living

1) Agenda Setting (1 min)
2) Safety Planning (5-10 min)
3) Values exploration (5 – 10 min)
4) Taking steps in the direction that matter (5-10 min)

Session 2: Building a life worth living

1) Welcome and opening housekeeping
   a. Hellos
   b. Tech check
2) Agenda Setting (1 min)
   For example: *'Before we dive in, I want to chat about the plan for today and get your input. First, I would love to touch base on the Safety Plan and if and how you have used that this week. Then, in the second session we typically zoom out a bit and talk big picture about building a life worth living. Then we'll discuss big-picture values, and think about how you want to start working toward those values."*
3) Safety Planning (5-10 min)
   -For example: "*Have you used your Safety Plan at all since we last talked?"*

   -If no: "*What got* in *the way of using it? Did* you *not have any thoughts about suicide* this week*?"*

   -Consider: "*Is there anything I can do to help make it easier for you to use the Safety Plan when you're in a crisis?"*
   1. Reflect the challenge they express. For example: "*You said that one reason you were hesitant to use the safety plan is because you feel like you are bothering people when you reach out for support. If you had some safety strategies that didn't lead you to feel like you are bothering people, the safety plan would feel more useful.*"
   2. Suggest troubleshooting together. For example: "*I am wondering if it might be helpful for us to troubleshoot this together, to think of some ways to get around the "I am bothering people" thought that gets in your way. Would you be willing to do that together?*"

      Use OARS to investigate the challenge. This part of the conversation will be responsive to what the participant says. Use combinations of OARS to identify the challenges and potential solutions to develop in the next section. Some examples of things you might say: "*Which of the strategies on your safety plan lead to the "I am bothering people" thoughts?...Which strategies don't bring up those thoughts?"* (Open Ended Question)

"The "bother" thoughts come up most when you reach out to the family members on your list for help, but don't come up as much when you reach out to specific friends on your list." (Reflection)

"You are really creative about identifying other ways to keep yourself safe when the "bother" thoughts get in the way of some of these other strategies." (Affirmation)

"While the "bother" thoughts get in the way of you reaching out for help sometimes, you have used alternative coping strategies during those times to keep yourself safe. You know that you are most likely to get the "bother" thoughts when you consider reaching out to family members, which leaves you with fewer useful safety strategies than you would like to have." (Summary)

3. Brainstorm potential solutions. If needed, offer information/solutions from STARS content. Reflect change talk throughout. This part of the conversation will be responsive to what the participant says. Use combinations of OARS to draw out the participant's solutions. If needed, ask whether they would like you to offer one or two potential solutions for them to consider. Some examples of things you might say:

    "You are creative and know yourself best – I am curious if you already have some thoughts about solutions that would work for you in this situation." (Open ended question)

    "Your aunt is one exception to the family "bother" rule – you know that she would never see you that way. She is one family member you could keep on that section of your safety plan." (Reflection)

    "You really care about your family and that care gives you a lot of strength to keep yourself safe through these crisis moments." (Affirmation)

    "*You were able to come up with a number of new strategies to replace the ones that were blocked by the "bother" thoughts. Removing some family members from the safety plan and replacing them with your aunt makes that part of the plan feel more useful. And you were able to reinforce which strategies still work when "bother" thoughts do come up."* (Summary)

-If yes: "*I'm glad that you tried using it. How did it go?"*

-If good: "*That's great I'm glad it was helpful when you were in a crisis."'*

-If bad: *"I'm sorry to hear that it wasn't helpful. What do you think prevented it from helping you?"*

1. Reflect both the partial success (they used it) and the challenge they express.
2. Suggest troubleshooting together.
3. Use OARS to investigate the challenge
4. Use OARS to brainstorm potential solutions. If needed, offer information/solutions from STARS content. Reflect change talk throughout.

-Inquiring about whether any edits should be made to it

-For example: "*Should we make any changes to the Safety Plan?"*

4) Values exploration (5 – 10 min)

-For example: *"So, today we want to talk a bit about values and ways we can use values to guide how we steer our lives in directions we find meaningful. Whenever I talk with people about values, particularly people who come historically marginalized backgrounds, whether because of gender, sexuality, race, disability, immigration status, or other parts of their identity, I think it is important to start by acknowledging that marginalization we have experienced may have influenced how we developed our values and the level of power we feel over them. We get many of our values from the people and communities that are important to us. And it's possible that we couldn't express some of our values until we began to gain independence as adults. That can leave some people feeling like they are still figuring out their values, both what they bring from their past and what they choose to pursue now. I am curious how much you feel that applies to you?*

[Brief reflective listening]

When you completed the baseline survey for the study, one of the questionnaires that you completed was called the Valued Living Questionnaire. This questionnaire first asked you to rate the importance of 10 life domains and then to rate how consistently you feel you are living with that domain. This allowed for us to calculate a difference score, where we subtract how consistently you are living your life with how important the area is. I've got those scores here – would you be okay with us looking at them together as a way to ground our conversation in some concrete numbers? [Review scores]. What stands out to you as we review these together?

[Reflective listening]

Now that we have talked a bit about your values, if you had to choose a few that are high-priority to you at this stage of your life, which would they be?

[Reflective listening]

Reviewing this questionnaire can be a helpful jumping off point for thinking about what matters to you and how you want to live your life.

*A **value** is something that you care about. You can never 'check-off' a value and say that you never have to work on it again. It's like a direction in life, almost like saying 'I'm heading East.' We learn some values from important people in our life, but you get to choose if you actually want to have something as a value. Just because it's important to other people doesn't mean you need to care about it. There's a reminder about values in the Explore article called Who am I?*

*In contrast, a **goal** is a marker along the way. If the value is the direction, the goal are the benchmarks that tell you if you are headed East or West.*

As simple as this sounds, talking about values be painful at times. Sometimes when we start talking about values, we start realizing where our lives are inconsistent with the values we hold. Not to worry—this is part of the process of building a life worth living. It's essential to reflect on where you are now, where YOU want to head, and the supports you have access to that can help you get there.

Any questions about that?

As I said at the beginning, talking about values, while it can be helpful and meaningful, can also be challenging. There are ways that the systems we live in make it challenging for us to express and pursue our values sometimes. And sometimes we get in our own way too – it takes purpose and energy to live in accordance with our values on a daily basis. I find some of the keys are just taking the time to remember what you value, why it's worth the effort, and not beating yourself up too much on your "cheat days."

Before we move on, what are some big picture values that you're interested in making progress on in the coming weeks?"

5) Taking steps in the direction that matter (5-10 min)

For example: "*It can be helpful to pair our values with goals, which, if values are the direction we want to go, goals are like signposts that tell you if you are headed the right way.*

I am curious about whether you might already have some existing goals related to [value that the participant mentioned] you are interested in making progress on? What, if any, challenges have you encountered with goals related to [value that the participant mentioned]?

[Reflective listening]

There are some common pitfalls that people run into (also discussed in the Making a Change article). Would you be interested in looking at some of them together?

If so: "For instance, it's really common for folks to set goals that are way too ambitious, often out of anxiety about feeling like 'I NEED TO DO MORE!!!!' That's a recipe for disaster because you'll miss the goal, and then feel worse. As another example, it's really common for folks to set goals that are too vague. The problem with vague goals it that you never know if you achieve them, so you never get to celebrate.

Given this, what are some goals that you're interested in setting? How will you know if you've met them?"

6) Summarize and close

- Close with brief summary, particularly highlighting any of the following that occurred
  - Any generated motivations to use the safety plan
  - Acknowledging changes made to safety plan and express interest in feedback as they try out the changes
  - Any substantive feedback they provided about the session
- Mention next week's topic (Dealing with negative self-talk)
- Thanks and closing

Session 3: Dealing with negative self-talk

1) Agenda Setting (1 min)
2) Safety Planning (5-10 min)
3) How thoughts impact our emotions (5 min)
4) Strategies for managing thoughts differently (10 min)
    a. Cognitive restructuring (changing thoughts to be more helpful)
    b. Cognitive defusion (getting distance from thoughts)

Session 3: Dealing with negative self-talk

1) Agenda Setting (1 min)

    For example: "*As usual, we'll start by checking in on the safety plan. Then we're going to discuss how negative self-talk can impact our emotions and our behaviors and talk about some strategies for managing these thoughts in a different way. How does that sound? Anything to add to the agenda?"*

2) Safety Planning (5-10 min)

    -For example: "*Have you been using your Safety Plan at all since we last talked?"*

    -If no: "*What got in the way of using it? Did you not have any thoughts about suicide this week?"*

    -If the person had suicidal thoughts but did not use the Safety Plan, consider: "*Is there anything I can do to help make it easier for you to use the Safety Plan when you're in a crisis?"*

    1. Reflect the challenge they express. For example: "*You said that one reason you were hesitant to use the safety plan is because you feel like you are bothering people when you reach out for support. If you had some safety strategies that didn't lead you to feel like you are bothering people, the safety plan would feel more useful.*"
    2. Suggest troubleshooting together. For example: "*I am wondering if it might be helpful for us to troubleshoot this together, to think of some ways to get around the "I am bothering people" thought that gets in your way. Would you be willing to do that together?*"

        Use OARS to investigate the challenge. This part of the conversation will be responsive to what the participant says. Use combinations of OARS to identify the challenges and potential solutions to develop in the next section. Some examples of things you might say: "*Which of the strategies on your safety plan lead to the "I am bothering people" thoughts?...Which strategies don't bring up those thoughts?"* (Open Ended Question)

        "The "bother" thoughts come up most when you reach out to the family members on your list for help, but don't come up as

much when you reach out to specific friends on your list." (Reflection)

"You are really creative about identifying other ways to keep yourself safe when the "bother" thoughts get in the way of some of these other strategies." (Affirmation)

"While the "bother" thoughts get in the way of you reaching out for help sometimes, you have used alternative coping strategies during those times to keep yourself safe. You know that you are most likely to get the "bother" thoughts when you consider reaching out to family members, which leaves you with fewer useful safety strategies than you would like to have." (Summary)

3. Brainstorm potential solutions. If needed, offer information/solutions from STARS content. Reflect change talk throughout. This part of the conversation will be responsive to what the participant says. Use combinations of OARS to draw out the participant's solutions. If needed, ask whether they would like you to offer one or two potential solutions for them to consider. Some examples of things you might say:

    "You are creative and know yourself best – I am curious if you already have some thoughts about solutions that would work for you in this situation." (Open ended question)

    "Your aunt is one exception to the family "bother" rule – you know that she would never see you that way. She is one family member you could keep on that section of your safety plan." (Reflection)

    "You really care about your family and that care gives you a lot of strength to keep yourself safe through these crisis moments." (Affirmation)

    "*You were able to come up with a number of new strategies to replace the ones that were blocked by the "bother" thoughts. Removing some family members from the safety plan and replacing them with your aunt makes that part of the plan feel more useful. And you were able to reinforce which strategies still work when "bother" thoughts do come up."* (Summary)

-If yes: "*I'm glad that you tried using it. How did it go?"*

-If good: "*That's great I'm glad it was helpful when you were in a crisis."'*

-If bad: *"I'm sorry to hear that it wasn't helpful. What do you think prevented it from helping you?"*

-Inquiring about whether any edits should be made to it

-For example: "*Should we make any changes to the Safety Plan?"*

3) How thoughts impact our emotions (5 min)

For example: *"Every emotion has three parts to it—thoughts, behaviors and physical sensations. Each of these three parts relates to the other, so that what you think influences what you do and how you physically feel, and all of these influence how you emotionally feel. A model that describes this is presented as an article in the App. A lot of times when we're feeling strong emotions, we have really intense and negative thoughts run through our mind—sometimes we might not even be aware of that. Even when we don't pay much attention to them, these extreme thoughts can tank our mood and make us feel terrible about ourselves, other people, or the world. [Mention the negative self-talk video on the app].*

One important first-step that you can take to loosen the grip that these thoughts have over you is to begin tracking what goes through your mind when you're feeling a strong emotion. There's an activity to help with this. For instance, in the past week, was there a time when you felt a strong, negative emotion? It could be anything (shame, guilt, sadness, anxiety, etc.). What was going through your mind when you felt that? In other words, what were you telling yourself?

4) Strategies for managing thoughts differently (10 min)

For example: "*In addition to becoming more aware of the impact of negative thoughts on our mood, it can also help to practice some strategies to respond to these thoughts differently. We have a few options for how to do that, both of which are in the app. The first one uses a tool called cognitive restructuring, or changing thoughts to be more helpful. The second uses a tool called cognitive defusion, or getting distance from thoughts. Which of these would you like to talk about today?"*

a. Cognitive restructuring (changing thoughts to be more helpful)

For example: "*There are three questions that it can be helpful to ask yourself about strong negative thoughts, namely: 1) Is it accurate?; 2) Is it specific (vs. global)?; and 3) Is it helping?*

When we're assessing accuracy, the question here is: 'Are there any other alternative possibilities?' For example, earlier you told me about the strong negative thought [enter thought here] when you were feeling [emotion]. What do you think: Was that thought accurate?

If relevant: *In a lot of cases, strong negative thoughts take the form of mind reading. Because you'll never know for sure what someone is thinking, mindreading thoughts can't be accurate.*

When we're assessing specificity, the question here is: 'Is this thought specific to the situation at hand, or has it ballooned out to be about more general things in my life?' An easy way to tell if a thought is specific is to assess whether it includes ***always*** *and* ***never*** *language. When we're depressed or anxious, we tend to 'extrapolate beyond the data' or make bigger meaning of smaller issues. Thus, it's important to track specificity.*

When we're assessing helpfulness, the question here is: 'Is this thought making it easier or harder for me to get my needs met in this moment?' To answer this thought, you have to know what is your need (sometimes we call this your 'desired outcome.') Once you identify what you're hoping will happen, then it's possible to assess whether the thought it taking you closer or further from your desired outcome.

In many cases, thoughts have components of accuracy (they may not be fully accurate), they may be specific, but they are often unhelpful. Some thoughts have all three problematic traits (not accurate, not specific and not helpful). Those are the doozy thoughts. Once you have identified what kind of thought you're dealing with, then you can ask: 'How can I edit this thought so that it is accurate, specific, and helping me get my desired outcome?'

Often the reframed thought sounds something like: 'If I want [X], I need to tell myself [Y].' For example: 'If I want to ask John to hang out, I need to remind myself of the things about me that are like-able, like that I'm kind, thoughtful, and funny.' Let's really highlight this for a minute. Imagine the impact of a thought like 'I'm kind, thoughtful and funny' on the likelihood of asking John to hang out. Now imagine the impact of a thought like 'He will never want to spend time with me because I don't have anything interesting to say.' These thoughts have important differences on what you choose to do.

b. Cognitive defusion (getting distance from thoughts)

For example: *"For some people, it can be helpful to remember that thoughts are just noise that our brain pumps out. You know how sometimes you might feel hungry even after you just ate? You might say to yourself 'Why am I hungry again???' Our bodies do weird things. So do our brains! Unfortunately, many of us treat the thoughts that our brain pumps out as super important.*

What if instead of assuming that your thoughts were really important and demand our attention, you assumed that your thoughts are just noise. You can think of it like the way that computers sometimes pump our random errors and silliness. Our brains do the same thing! The difference is that sometimes we treat the noise as facts and let it dictate what we do. For instance, having the thought

'I'm unlovable' might make you engage in behaviors that pull you away from other people. That's an awful consequence of brain noise.

There are a few strategies you can try to get distance from these thoughts so that they aren't so 'sticky' and don't hold so much weight over you. These are included in the articles on the app.

One strategy is called 'I'm having the thought that.' The basic idea is that you can preface any strong thought with 'I'm having the thought that' to remind yourself that the thought is just that—a thought—and a product of your brain. So for instance, if you find yourself having a really negative thought about yourself, like 'I'm such a loser and I'll never make any friends.' This would become 'I'm having the thought that I'm such a loser and I'll never make any friends.' Using this strategy offers you and opportunity to get some distance from the thought, by recognizing it as just a thought. It may also allow for you to build some empathy for yourself and to see 'Wow, I am really hard on myself. I have to practice being kinder to myself.'

A second strategy is called the 'and/but' exercise. This is relevant to thoughts that you have that include a 'but.' For instance, 'I want to ask John to hang out, but I'm nervous.' Change the but to an and, so that the new thought is: 'I want to ask John to hang out ***and*** *I'm nervous.' It is possible to feel negative emotions and do thing that we care about (things that make our life worth living). Sometime, the noise from our brain tricks us into thinking 'I can't do that BECAUSE I'm nervous.'*

Summarize and close

- Close with brief summary, particularly highlighting any of the following that occurred
    - Any generated motivations to use the safety plan
    - Acknowledging changes made to safety plan and express interest in feedback as they try out the changes
    - Any substantive feedback they provided about the session
- Mention next week's topic (Building positive experiences)
- Thanks and closing

Session 4: Working to build positive experiences in your life

1) Agenda Setting (1 min)
2) Safety Planning (5-10 min)
3) Building positive experiences (10 min)
    a. Scheduling pleasant activities
    b. Rewarding yourself for meeting your goals
    c. Savoring pleasant moments

Session 4: Working to build positive experiences in your life

1) Agenda Setting (1 min)

    -For example: "*Today we're going to briefly check in on your Safety Plan and then spend the rest of our time talking about ways to build positive emotions. Does that sound okay to you? Anything you want to add to the agenda?"*

2) Safety Planning (5-10 min)
    -For example: "*Have you been using your Safety Plan at all since we last talked?"*

    -If no: "*What got in the way of using it? Did you not have any thoughts about suicide this week?"*

    -If the person had suicidal thoughts but did not use the Safety Plan, consider: "*Is there anything I can do to help make it easier for you to use the Safety Plan when you're in a crisis?"*

    1. Reflect the challenge they express. For example: "*You said that one reason you were hesitant to use the safety plan is because you feel like you are bothering people when you reach out for support. If you had some safety strategies that didn't lead you to feel like you are bothering people, the safety plan would feel more useful.*"
    2. Suggest troubleshooting together. For example: "*I am wondering if it might be helpful for us to troubleshoot this together, to think of some ways to get around the "I am bothering people" thought that gets in your way. Would you be willing to do that together?*"

        Use OARS to investigate the challenge. This part of the conversation will be responsive to what the participant says. Use combinations of OARS to identify the challenges and potential solutions to develop in the next section. Some examples of things you might say: "*Which of the strategies on your safety plan lead to the "I am bothering people" thoughts?...Which strategies don't bring up those thoughts?"* (Open Ended Question)

        "The "bother" thoughts come up most when you reach out to the family members on your list for help, but don't come up as

much when you reach out to specific friends on your list." (Reflection)

"You are really creative about identifying other ways to keep yourself safe when the "bother" thoughts get in the way of some of these other strategies." (Affirmation)

"While the "bother" thoughts get in the way of you reaching out for help sometimes, you have used alternative coping strategies during those times to keep yourself safe. You know that you are most likely to get the "bother" thoughts when you consider reaching out to family members, which leaves you with fewer useful safety strategies than you would like to have." (Summary)

3. Brainstorm potential solutions. If needed, offer information/solutions from STARS content. Reflect change talk throughout. This part of the conversation will be responsive to what the participant says. Use combinations of OARS to draw out the participant's solutions. If needed, ask whether they would like you to offer one or two potential solutions for them to consider. Some examples of things you might say:

    "You are creative and know yourself best – I am curious if you already have some thoughts about solutions that would work for you in this situation." (Open ended question)

    "Your aunt is one exception to the family "bother" rule – you know that she would never see you that way. She is one family member you could keep on that section of your safety plan." (Reflection)

    "You really care about your family and that care gives you a lot of strength to keep yourself safe through these crisis moments." (Affirmation)

    "*You were able to come up with a number of new strategies to replace the ones that were blocked by the "bother" thoughts. Removing some family members from the safety plan and replacing them with your aunt makes that part of the plan feel more useful. And you were able to reinforce which strategies still work when "bother" thoughts do come up."* (Summary)

-If yes: "*I'm glad that you tried using it. How did it go?"*

-If good: "*That's great I'm glad it was helpful when you were in a crisis."'*

-If bad: *"I'm sorry to hear that it wasn't helpful. What do you think prevented it from helping you?"*

-If edits are indicated, jump ahead to edits section below.

-Inquiring about whether any edits should be made to it

-For example: "*Should we make any changes to the Safety Plan?*"

3) Building positive experiences (10 min)
   a. Scheduling pleasant activities
      i. Elicit activities that make the person feel accomplished

         -For example: "*Research shows that one of the best ways to build positive emotions is to practice noticing what activities make you feel more accomplished. Maybe we could start by brainstorming some activities that make you feel accomplished or proud of yourself. Do you have any examples of those?"*

      ii. Elicit activities that make the person feel more enjoyment.

         -For example: "*It's also important to keep track of the kinds of things that give you a sense of enjoyment or pleasure (and that don't backfire in harmful ways later). Do you have any examples of those?"*

      iii. Scheduling accomplishment-based or enjoyable activities.

         -For example: "*It's one thing to know what activities make you feel more accomplished/more enjoyment, but it's another thing to commit to doing them. Would you be willing to schedule in some activities that are likely to give you a sense of accomplishment this week? When would you like to do them? How would you like to remind yourself of them? You can click on* ***GOALS*** *to track them in the app."*

      iv. Determining commitment

         -For example: "*On a scale from 0 – 100% (where 100% is completely confident and 0% is it's not going to happen), how confident are you that you will complete those activities?"*

         -If < 80%: "*Is there anything we could do to make it more likely that you can do these activities, even if it means making the activity a little easier this time? I'd rather you set something that you're confident you can do and slowly build up to bigger goals for yourself—that's the way to set yourself up for success."*

   b. Rewarding yourself for meeting your goals
      i. Psychoeducation about the importance of rewards

         -For example: *"It's really common for people to jump from one goal to the next without ever celebrating their victories. This is so boring!!!!!! It makes life a slog. It also makes it hard to sustain—eventually if you keep pushing and pushing without celebrating, you will burnout and want to quit altogether. To break that cycle, it is EXTREMELY important to reward*

*yourself for your own good behavior. If you don't, who will? (*This is rhetorical, obvi.)"

-Note: Some people will react to this idea by saying "I shouldn't have to reward myself for doing things I have to do." A common response I use to this is: "*Where did you learn that it's bad to reward yourself? Says who? Would you do a job for free? Hell no! So why is this different?"* You might find that you need frequent reminders about this toxic mentality throughout the sessions.

ii. Planning rewards

-For example: "*Given that you set some new goals for this week, how can you reward yourself for them?"*

iii. Linking rewards to low-likelihood tasks

-For example: "*A last point about rewards is that they can be so helpful at trying to change what we call 'low-likelihood behaviors.' There's this idea in psychology call the Premack principle. The idea is that we all have high-likelihood behaviors (these are things we are likely to do, like binge-watch Netflix after a long day). We also have low-likelihood behaviors (these are things that were aren't as likely to do, like cooking a healthy meal or having a difficult phone call). You can use high-likelihood behaviors as rewards for low-likelihood behaviors! For instance, if you need to call someone to have a difficult conversation but you've been putting it off, consider a plan to reward yourself with your favorite show immediately after the call (and commit to not watching the show until the call happens). Does that make sense?"*

c. Savoring pleasant moments (for this section, you should present the psychoeducation and then give the option for which tools to discuss as you won't have time for all).

i. Psychoeducation about why it's important to savor the moment

-For example: "*We often go through life in a flash. We might have to race to get out of the apartment, run to catch the bus, and skip lunch just to make it from one gig to the next. This is exhausting. It also means that we are often primed to be planning ahead rather than staying in the present moment. Research shows that when we are thinking about the future, we tend to feel anxious. When we are thinking about the past, we tend to feel depressed. The goal for achieving mental balance is staying in the present moment, and bringing yourself back to the present moment when you catch yourself jumping to the future or past. Staying in the present moment is super important when you're doing these achievement/enjoyment tasks. If you don't let yourself savor in the moment, you won't experience the benefit of the task."*

ii. Solicit input on how the participant would like to discuss this topic

-For example: "*We have many strategies to savoring pleasant moments in the app. Some examples include ways to boost present moment awareness, ways to describe positive experiences, ways to practice self-*

compassion and compassion for others, ways to practice harnessing gratitude, and ways to practice generosity to your community. Which of these would it be most helpful to discuss?"

iii. Tips and tricks for how to do this

-Present moment awareness

-For example: "*If you want to train yourself to saver the pleasant moments, practice redirecting your attention back to the present. Every time you notice yourself planning or judging or thinking about the past, catch yourself and gently redirect back to the here-and-now. You might say to yourself 'There I go again' as you gently bring your attention back. Redirecting your attention is like building a muscle—it gets easier over time, but it takes a lot of practice. Don't be hard on yourself, that will only make you feel worse."*

-Describe the pleasant/pride boosting experience

-For example: "*Label what about the experience is bring you joy or making you feel pride. Even taking a moment to label it to yourself can help you to savor it."*

-Practice compassion

-For example: "*Some people were taught that it's bad to be proud or that they don't deserve to enjoy themselves. Other people just think that way even if no one explicitly taught them this message. If you struggle with this, you might consider some self-compassion mindfulness activities, Do you know how to find examples of those?"*

-Gratitude building

-For example: "*Sometimes it is easy to get stuck on all of the things that have gone wrong in our lives, which makes us feel more depressed. While you have every right to feel hurt and disappointed by what has gone wrong, it is also important to acknowledge what has gone right. Getting anchored on the things for which we feel grateful can help bring us into the present moment and savor positive experiences in the future. To do this, you can make a list of the things in your life that you feel grateful for now. For these, you can click on the Activity called "**GRATITUDE LIST**."*

Summarize and close

- Close with brief summary, particularly highlighting any of the following that occurred
    - Any generated motivations to use the safety plan

    - Acknowledging changes made to safety plan and express interest in feedback as they try out the changes
    - Any substantive feedback they provided about the session
- Mention next week’s topic (Dealing with people who are hurting you)
- Thanks and closing

Session 5: Dealing with people who are hurting you

1) Agenda Setting (1 min)
2) Safety Planning (5-10 min)
3) Orientation to module (2 min)
4) Recognizing discrimination (5 min)
5) Options for discussion (5-10 min)
6) Distress tolerance (5 min)
7) Essentials (5 min)

Session 5: Dealing with people who are hurting you

1) Agenda Setting (1 min)

   For example: *"Today we're going to briefly check in on your Safety Plan and then spend the rest of our time talking about ways to support yourself in the face of discrimination. Does that sound okay to you? Anything you want to add to the agenda?"*

2) Safety Planning (5-10 min)

   For example: "*Have you been using your Safety Plan at all since we last talked?"*

   -If no: "*What got in the way of using it? Did you not have any thoughts about suicide this week?"*

   -If the person had suicidal thoughts but did not use the Safety Plan, consider: "*Is there anything I can do to help make it easier for you to use the Safety Plan when you're in a crisis?"*

   1. Reflect the challenge they express. For example: "*You said that one reason you were hesitant to use the safety plan is because you feel like you are bothering people when you reach out for support. If you had some safety strategies that didn't lead you to feel like you are bothering people, the safety plan would feel more useful.*"
   2. Suggest troubleshooting together. For example: "*I am wondering if it might be helpful for us to troubleshoot this together, to think of some ways to get around the "I am bothering people" thought that gets in your way. Would you be willing to do that together?*"

      Use OARS to investigate the challenge. This part of the conversation will be responsive to what the participant says. Use combinations of OARS to identify the challenges and potential solutions to develop in the next section. Some examples of things you might say: "*Which of the strategies on your safety plan lead to the "I am bothering people" thoughts?...Which strategies don't bring up those thoughts?"* (Open Ended Question)

      "The "bother" thoughts come up most when you reach out to the family members on your list for help, but don't come up as

much when you reach out to specific friends on your list." (Reflection)

"You are really creative about identifying other ways to keep yourself safe when the "bother" thoughts get in the way of some of these other strategies." (Affirmation)

"While the "bother" thoughts get in the way of you reaching out for help sometimes, you have used alternative coping strategies during those times to keep yourself safe. You know that you are most likely to get the "bother" thoughts when you consider reaching out to family members, which leaves you with fewer useful safety strategies than you would like to have." (Summary)

3. Brainstorm potential solutions. If needed, offer information/solutions from STARS content. Reflect change talk throughout. This part of the conversation will be responsive to what the participant says. Use combinations of OARS to draw out the participant's solutions. If needed, ask whether they would like you to offer one or two potential solutions for them to consider. Some examples of things you might say:

    "You are creative and know yourself best – I am curious if you already have some thoughts about solutions that would work for you in this situation." (Open ended question)

    "Your aunt is one exception to the family "bother" rule – you know that she would never see you that way. She is one family member you could keep on that section of your safety plan." (Reflection)

    "You really care about your family and that care gives you a lot of strength to keep yourself safe through these crisis moments." (Affirmation)

    "*You were able to come up with a number of new strategies to replace the ones that were blocked by the "bother" thoughts. Removing some family members from the safety plan and replacing them with your aunt makes that part of the plan feel more useful. And you were able to reinforce which strategies still work when "bother" thoughts do come up."* (Summary)

-If yes: "*I'm glad that you tried using it. How did it go?"*

-If good: "*That's great I'm glad it was helpful when you were in a crisis."'*

-If bad: *"I'm sorry to hear that it wasn't helpful. What do you think prevented it from helping you?"*

-Inquiring about whether any edits should be made to it

-For example: "*Should we make any changes to the Safety Plan?"*

3) Orientation to module (2 min)

For example: *"The focus for today is on strategies to deal with people who have hurt you through acts of racism, sexism, homophobia/transphobia, ageism, classism or other forms of hatred, or oppression.*

How are you experiencing discrimination right now or in the past?

The perspective that we are taking here is that it is completely unfair that you might have had to deal with any of these forms of oppression. Until the world becomes more welcoming and compassionate, it can be helpful to practice strategies to promote self-love so that the toxic nature of oppression does not eat away at you."

4) Recognizing discrimination (5 min)

For Example, "*Discrimination can pop up in sneaky ways or in really obvious ways. Let's spend a few minutes talking about recognizing discrimination. Also these messages are reflected in the Discrimination articles on the app. First, some common examples of discrimination include being avoided, not having equal rights, or being emotionally, physically, verbally or sexually attacked just because of who you are or how you look. Discrimination often begins because of a stereotype and can lead to bullying. Sometimes it's really obvious. Sometimes it's harder to recognize, like when a friend makes fun of you a lot but gas lights you by saying 'I'm just kidding!' Or someone says that they support you but don't talk to you about what you care about. Sometimes you might not have concrete examples of it, but you just have a gut feeling about being mistreated. These all could be indicators that you are experiencing discrimination.*

When you have noticed that you are experiencing discrimination, you have some options for things to do to help you feel less drained by the toxicity. For this module, it's a 'choose your own adventure day.' I'm going to present to you some options for what we can discuss, and you can choose which of these you'd like to dive into."

5) Options for discussion (each could be 5 – 10 min, and you will choose 1)
   a. Self-love

   For example: *"Dealing with hatred from the world can take a toll on our self-esteem. It's essential to practice building yourself up. If you don't, who will? There are a few key messages here, which are also reflected in the Dealing with Discrimination article. The first is to remind yourself that you do not deserve to be treated poorly. You 100% deserve to feel loved, cared for, and safe. To remind yourself of all of the wonderful things about you, it's usually helpful to start with taking an inventory (you can access this as an*

activity called Things I Love About Me). I'd like for you to open this up and make a list of the things that you like about yourself and the things that you are good at. What have other people complimented you on in the past? What things have you done that have brought joy to others? I'd like for us to take a few minutes to start this list. Then I'd like for you to come back to this sometime later this week to add more to it. The trick is making sure that the list is generous AND genuine. I'm not asking you to lie to yourself, but we tend to be naturally too hard on ourselves, so this is a time to practice building yourself up!"

b. Compassion

For example: "*It can be helpful to practice compassion for ourselves and for other people. There are a few key messages about how to do this, which are also reflected in the articles on self-compassion. Judgment is like anger; if you let it run rampant on others it will hurt you too, and if you use it on yourself it will hurt others. You might consider some* ***loving kindness meditations*** *to help build compassion for yourself and the world."*

c. Acceptance

For example: "*Serenity is all about balancing what you can change and what you need to accept. Acceptance isn't the same as resignation. It is embracing pain because you feel it whether or not you are willing to feel it. It is acknowledging the truth of the world and the reality that stigma is real and painful. Denial doesn't help. There may be things about your life that you can change to reduce how often you are stigmatized—for example, maybe there are hurtful people in your life with whom you no longer want to associate. If you want to make those changes, go for it! However, sometimes the sources of our pain cannot be changed. In those cases, acceptance can give you peace of mind that there is nothing more you can do in this moment, and you might as well tolerate the situation as it is. There are some radical acceptance exercises that you can practice to help you find peace with this. Have you ever done any of those? Do you know where to find them? Important note: By encouraging acceptance, I am not saying that you should tolerate people being mean to you or hurting you. Not at all. The reality is that no matter what you do or how effective you are, some people might still be jerks. Acceptance is about dropping the "wishful thinking" about wanting the situation to be different. This gives you more freedom to spend time on things that matter to you."*

6) Essentials of managing discrimination

For example: "In addition to all of these tips and tricks, there are some essentials to consider when you encounter discrimination. These include:

-Recognize when it happens and how it makes you feel

-If you can, remove yourself from the situation

-Make up an excuse like answering your phone, go somewhere safe, find someone who will support you

-Ignore rude comments if you can

-Try and change the topic or try using humor to make the conversation more positive

-Breathe and support yourself

-Take some deep breaths (longer exhales than inhales)

-Silently tell yourself what you'd want to hear from a friend ("I love you, support you, and you're great just as you are")

-If you're in a safe space and have the privilege to, call them out

-Firmly tell them to stop

-Tell them that you feel frustrated or mistreated

-Try to keep it from becoming a fight by avoiding being mean or rude

-Sometimes you have to tell people why things are offensive

-If things get violent, get help immediately

7. Distress tolerance

Sometimes when people or institutions are hurting us, it can really impact our emotions. The problem is, when our emotions are super intense (a "10 out of 10", where 10 is as upset as you could possibly be), it is hard to be as effective as we would want. In those moments, the goal is to bring down emotional intensity so that it's less overwhelming as so that it's more likely to survive the situation.

We often recommend an Emotional Disaster Kit, or distress tolerance kit, as a way to bring down emotional intensity. This kit is often a physical kit that includes ways to get in touch with senses. Everyone's kit looks different. It can include your favorite scented lotions, candles, bubble bath mix, nail polish, bubble wrap to pop, fabric to feel, images of beautiful pictures, attention-grabbing poetry, a playlist of de-escalating music, or whatever works for you.

Would it be helpful to you if we were to brainstorm some things that could go in your distress tolerance kit? Summarize and close

- Close with brief summary, particularly highlighting any of the following that occurred
  - Any generated motivations to use the safety plan
  - Acknowledging changes made to safety plan and express interest in feedback as they try out the changes
  - Any substantive feedback they provided about the session
- Mention next week's topic (Managing Relationships)
- Thanks and closing

Session 6: Managing relationships

1) Agenda Setting (1 min)
2) Safety Planning (5-10 min)
3) Organizations and support groups (5-10 min)
4) Getting the support you need (5-10)
5) How to be supportive (5-10)

Session 6: Managing relationships

1) Agenda Setting (1 min)
2) Safety Planning (5-10 min)
   For example: *"Have you been using your Safety Plan at all since we last talked?"*

   -If no*: "What got in the way of using it? Did you not have any thoughts about suicide this week?"*

   -If the person had suicidal thoughts but did not use the Safety Plan, consider: *"Is there anything I can do to help make it easier for you to use the Safety Plan when you're in a crisis?"*

   1. Reflect the challenge they express. For example: "*You said that one reason you were hesitant to use the safety plan is because you feel like you are bothering people when you reach out for support. If you had some safety strategies that didn't lead you to feel like you are bothering people, the safety plan would feel more useful.*"
   2. Suggest troubleshooting together. For example: "*I am wondering if it might be helpful for us to troubleshoot this together, to think of some ways to get around the "I am bothering people" thought that gets in your way. Would you be willing to do that together?*"

      Use OARS to investigate the challenge. This part of the conversation will be responsive to what the participant says. Use combinations of OARS to identify the challenges and potential solutions to develop in the next section. Some examples of things you might say: "*Which of the strategies on your safety plan lead to the "I am bothering people" thoughts?...Which strategies don't bring up those thoughts?"* (Open Ended Question)

      "The "bother" thoughts come up most when you reach out to the family members on your list for help, but don't come up as much when you reach out to specific friends on your list." (Reflection)

      "You are really creative about identifying other ways to keep yourself safe when the "bother" thoughts get in the way of some of these other strategies." (Affirmation)

"While the "bother" thoughts get in the way of you reaching out for help sometimes, you have used alternative coping strategies during those times to keep yourself safe. You know that you are most likely to get the "bother" thoughts when you consider reaching out to family members, which leaves you with fewer useful safety strategies than you would like to have." (Summary)

3. Brainstorm potential solutions. If needed, offer information/solutions from STARS content. Reflect change talk throughout. This part of the conversation will be responsive to what the participant says. Use combinations of OARS to draw out the participant's solutions. If needed, ask whether they would like you to offer one or two potential solutions for them to consider. Some examples of things you might say:

   "You are creative and know yourself best – I am curious if you already have some thoughts about solutions that would work for you in this situation." (Open ended question)

   "Your aunt is one exception to the family "bother" rule – you know that she would never see you that way. She is one family member you could keep on that section of your safety plan." (Reflection)

   "You really care about your family and that care gives you a lot of strength to keep yourself safe through these crisis moments." (Affirmation)

   "*You were able to come up with a number of new strategies to replace the ones that were blocked by the "bother" thoughts. Removing some family members from the safety plan and replacing them with your aunt makes that part of the plan feel more useful. And you were able to reinforce which strategies still work when "bother" thoughts do come up."* (Summary)

-If yes: *"I'm glad that you tried using it. How did it go?"*

-If good: *"That's great I'm glad it was helpful when you were in a crisis."'*

-If bad: *"I'm sorry to hear that it wasn't helpful. What do you think prevented it from helping you?"*

-Inquiring about whether any edits should be made to it

-For example: *"Should we make any changes to the Safety Plan?"*

3) Organizations and support groups (5 – 10 min)

-For example: *"There are tons of resources in the app for getting connected to organizations and support groups here hmp://carelocator. One great option is CenterLink which you can use to find a center that you like for local meetings or other resources. Another great option is PFLAG.org, which has tons of amazing resources. There's also the American Foundation for Suicide Prevention which has a really great website and a very active local chapter.*

Would you ever consider joining a support group? If you decide to go to a support group, what are some factors you might consider? (If they don't have any ideas) Here are some things to consider:

-Decide whether you want to go alone
-You or your family may consider sharing your story with the group
-Ask questions
-Get to know other group members

Are there any organizations or support groups that you would like to get linked to? What would make that harder or easier for you? How can I help you think through your options or goals for this?

4) Getting the support you need

For example: "*It can be so difficult to reach out and ask for help. And yet, not asking for help can often make people feel more alone or even more suicidal..*

Who do you go to when you need support?

What makes it easier or harder to go to that person/those people?

-What do you need to remind yourself of to make it easier to muster the courage to ask for support when you need it?

-Remember that asking for help sooner can stop a crisis from ever starting, which will reduce the chaos in your life overall. It's harder to ask for help now (because it can be embarrassing or shameful) but it's easier in the long run because you get the help you need!

Just like how your Safety Plan is laid out, not all people in your life can serve all roles for you as it relates to support. Some people are awesome at just distracting you because they tell hilarious stories or are so self-focused that it takes the attention off of you (we all have those people in our lives). Other people are awesome and safe to open up to, even about really painful stuff—they won't judge you or make you feel dumb. Who are those people in your life?

Do you feel like you need more people in your life? Do you have any ideas about what you can do to expand your social circle?"

5) Setting boundaries/making a request

"A lot of people struggle with setting boundaries or making requests of other people, especially if they are already feeling lonely or like a burden on others. Some people have a hard time speaking up for themselves, whereas others accidentally come across as too intense when they were just trying to be assertive. Is this something that you ever struggle with?

If yes: *"There's a skill that's made for handling this. It's called the DEAR MAN skill and it comes from DBT (Dialectical Behavior Therapy). Would it be helpful if I teach you the skill?*

The 'DEAR' part is the acronym to remember what you say. The 'MAN' part is the acronym to remember how you say it.

What you say

D – Describe

This is BRIEFLY (1-2 sentences, maximum) describing the context for why you are making a request or setting a boundary. You can think about this like setting yourself up for a smooth landing. It's only what the participant needs to know to understand why you are making the request or setting the boundary—no more, no less.

E – Express

This is the (ideally vulnerable) emotion underlying the request/boundary. If anger is the only genuine emotion, it's fine to share that this is making you angry. However, anger tends to push people away—it's more effective to share more vulnerable emotions, like feeling hurt, afraid, sad or embarrassed if they are true.

A – Assert

This is when you actually make the request in 1 sentence. Always do this after describe and express.

R – Reinforce

How could it actually be a good thing for the other person if they meet your need/respect your boundary? Often it's the trickiest to think of this one, but there is always a silver lining. For instance, if you say 'No' to this request, maybe it will make you have more energy to spend time with the person later in the week, or be more available to be a good friend in general, as an example.

How you say it

M – Mindful

Even if you have 10 requests to make, focus on just the most important one. If the audience brings up something irrelevant, find a way to gently redirect the conversation back to your request (e.g., 'I'm hearing that's important to you, but I'd like for us to figure out [my issue] before we circle back to that.')

A – Appear confident

Think of a confident celebrity. What does their body language do to convey confidence? Practice emulating that until you find a style that works for you. This also means don't apologize for making a request (or for existing).

N - Negotiate

You have your preferred outcome (Plan A), but have backup options (Plan B and Plan C) in your mind so that you can negotiate and come to an arrangement that works for you and your audience.

6) How to be supportive

-For example: "*Every relation that we have is like a bank account. When we need something, we make a withdrawal of money. When we invest in the other person, it's like making a deposit. The trick is to make sure that you always have a balance so that there are 'available funds!'*

What are some things that you can do to invest in the people that you care about this week?

-It might seem overwhelming to focus on other people when YOU are the one that is struggling with feeling suicidal. However, research shows that investing energy into (healthy) other people can actually reduce the focus on us, which can reduce negative emotions. Believe it or not, it works!

Do you struggle to connect with people when you spend time with them? Here are some tips.

-Listen

-Be willing to talk honestly

-No one wants to talk to someone who always pretends things are always fantastic! (Though it's true that no one wants to talk to someone who only complains. It's all about the balance—if you make a withdrawal by having a complain day, make an investment next time by making it all about your friend!)

-Be patient with yourself and others

Summarize and close

- Close with brief summary, particularly highlighting any of the following that occurred
    - Any generated motivations to use the safety plan
    - Acknowledging changes made to safety plan and express interest in feedback as they try out the changes
    - Any substantive feedback they provided about the session
- Thanks and closing
    - *"It's been so meaningful to me to get to spend this time from you. I'm really impressed with [note personalized feedback on the courage, motivation, progress, or some other positive attribute of the participant]. I want to sincerely thank you for spending this time with me. I am so excited for your future and for you to accomplish all of the amazing things you are setting yourself up to do! Do you have any feedback for me?*
    - *I want to just reiterate that I've really enjoyed our time and I wish you the absolute best of luck as you work toward your goals."*
