# Supplementary figures and images for: A Suicide Prevention Intervention for Emerging Adult Sexual and Gender Minority Groups: Protocol for a Pilot Hybrid Effectiveness Randomized Controlled Trial
Source: JMIR Res Protoc. 2023 Sep 29;12:e48177. doi: 10.2196/48177 (PMC10576233; doi:10.2196/48177)

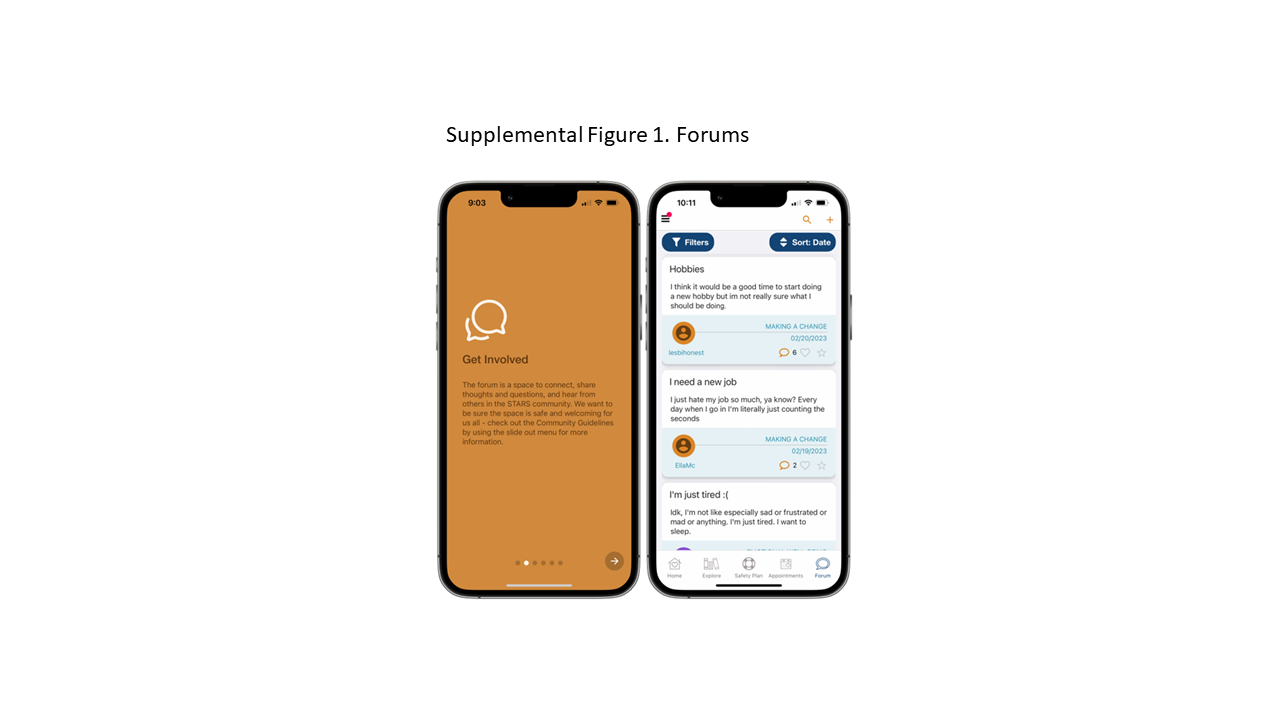

Supplement: Multimedia Appendix 2 [file resprot_v12i1e48177_app2.png]

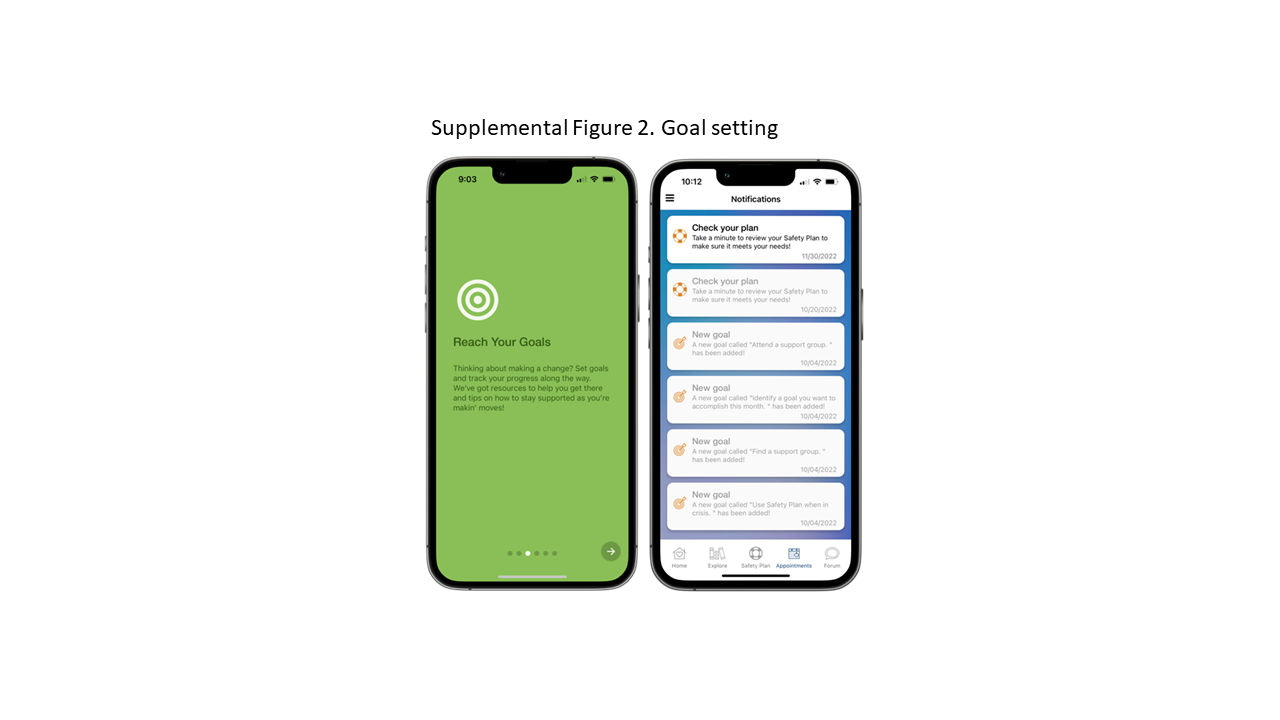

Supplement: Multimedia Appendix 3 [file resprot_v12i1e48177_app3.png]

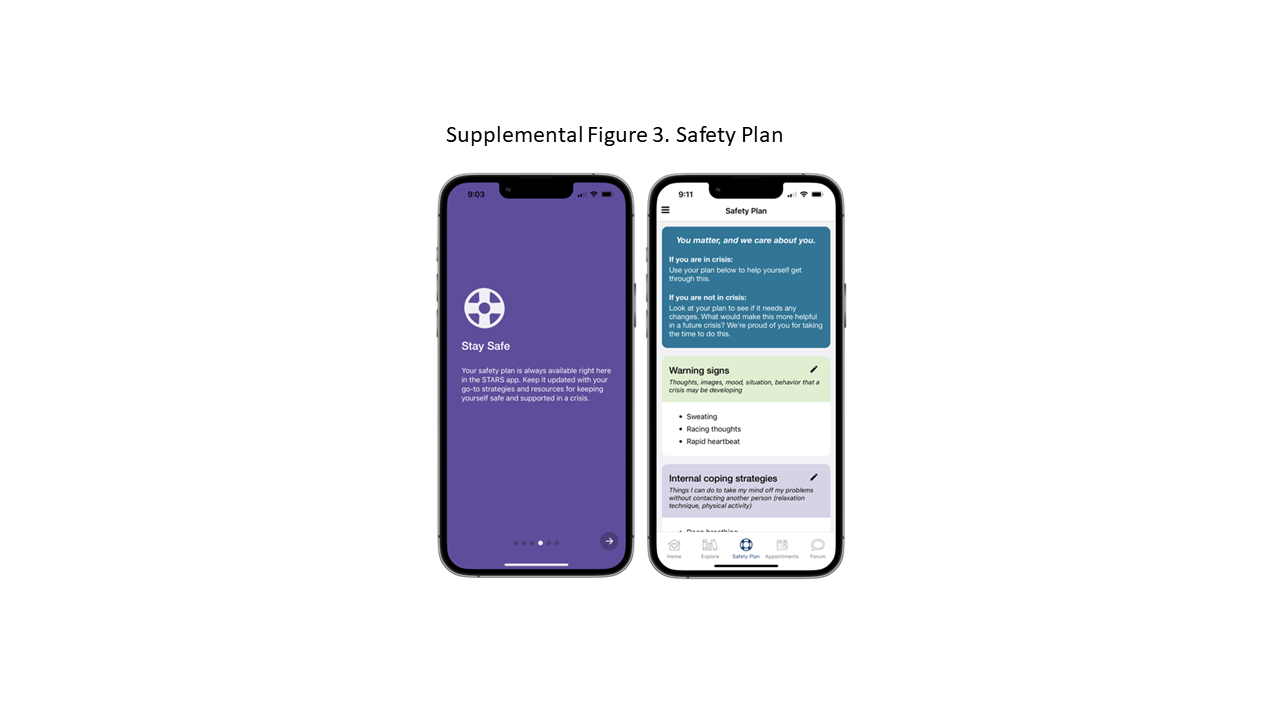

Supplement: Multimedia Appendix 4 [file resprot_v12i1e48177_app4.png]

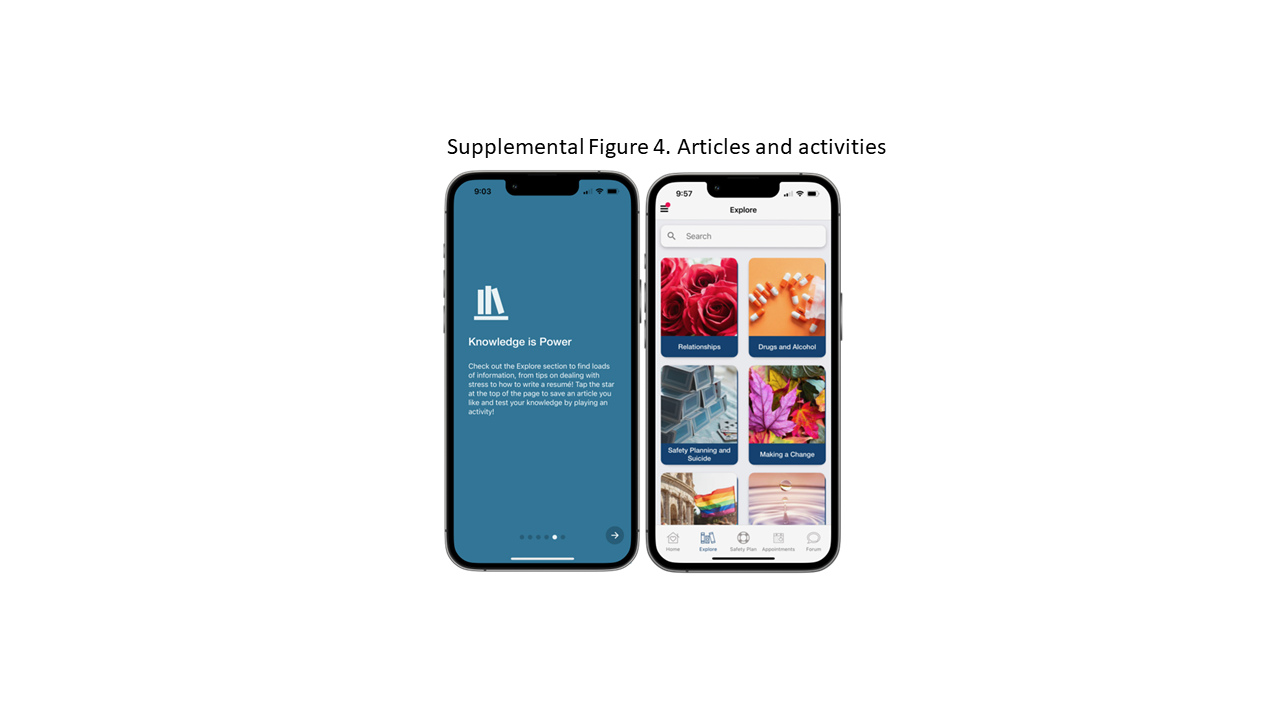

Supplement: Multimedia Appendix 5 [file resprot_v12i1e48177_app5.png]

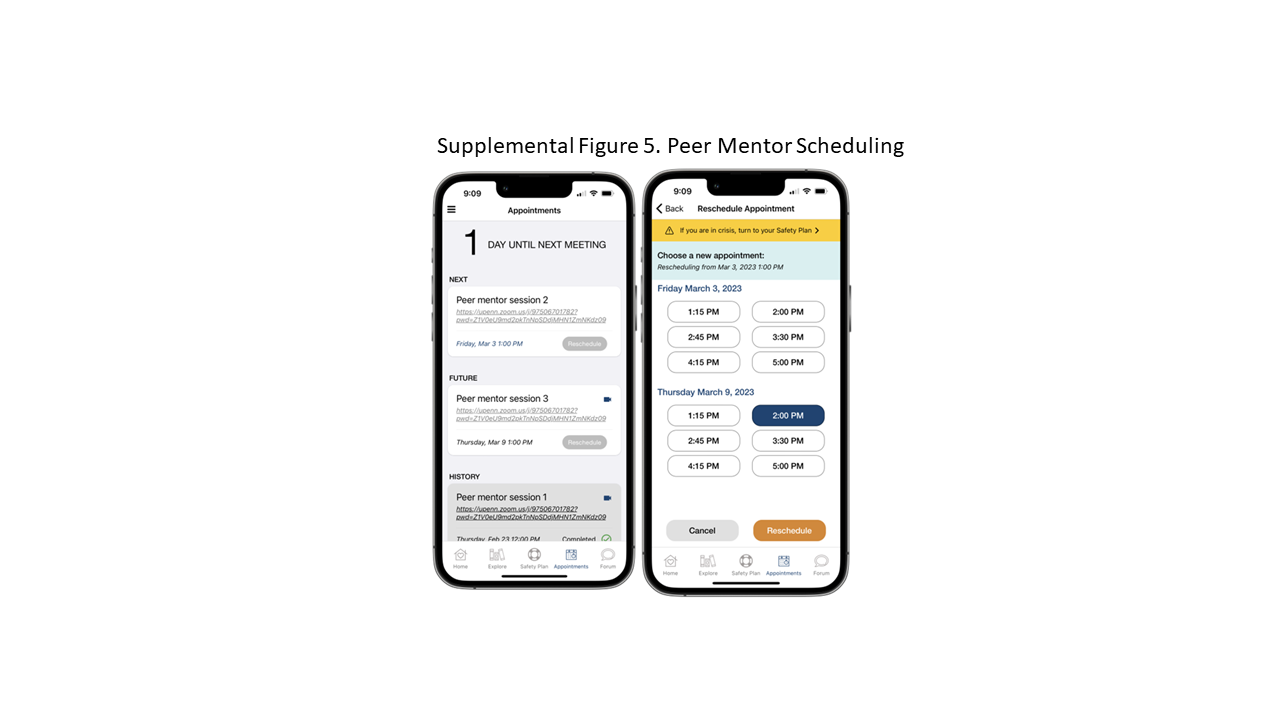

Supplement: Multimedia Appendix 6 [file resprot_v12i1e48177_app6.png]
